# High-sugar, high-fat, and high-protein diets promote antibiotic resistance gene spreading in the mouse intestinal microbiota

**DOI:** 10.1080/19490976.2021.2022442

**Published:** 2022-01-14

**Authors:** Rong Tan, Min Jin, Yifan Shao, Jing Yin, Haibei Li, Tianjiao Chen, Danyang Shi, Shuqing Zhou, Junwen Li, Dong Yang

**Affiliations:** Department of Environment and Health, Tianjin Institute of Environmental and Operational Medicine, Key Laboratory of Risk Assessment and Control for Environment & Food Safety, Tianjin, P. R. China

**Keywords:** Diet, antibiotic resistance gene, inflammatory microenvironment, amplification and transfer, pathogenic bacteria

## Abstract

Diet can not only provide nutrition for intestinal microbiota, it can also remodel them. However, is unclear whether and how diet affects the spread of antibiotic resistance genes (ARGs) in the intestinal microbiota. Therefore, we employed selected high-sugar, high-fat, high-protein, and normal diets to explore the effect. The results showed that high-sugar, high-fat, and high-protein diets promoted the amplification and transfer of exogenous ARGs among intestinal microbiota, and up-regulated the expression of *trfAp* and *trbBp* while significantly altered the intestinal microbiota and its metabolites. Inflammation-related products were strongly correlated with the spread of ARGs, suggesting the intestinal microenvironment after diet remodeling might be conducive to the spreading of ARGs. This may be attributed to changes in bacterial membrane permeability, the SOS response, and bacterial composition and diversity caused by diet-induced inflammation. In addition, acceptor bacteria (zygotes) screened by flow cytometry were mostly *Proteobacteria, Firmicutes* and *Actinobacteria*, and most were derived from dominant intestinal bacteria remodeled by diet, indicating that the transfer of ARGs was closely linked to diet, and had some selectivity. Metagenomic results showed that the gut resistance genome could be affected not only by diet, but by exogenous antibiotic resistant bacteria (ARB). Many ARG markers coincided with bacterial markers in diet groups. Therefore, dominant bacteria in different diets are important hosts of ARGs in specific dietary environments, but the many pathogenic bacteria present may cause serious harm to human health.

## 1. Introduction

The human gut is home to trillions of microorganisms and thousands of bacteria classes.^1^ There are six main categories and most are anaerobes.^2^ The number of bacteria in different regions of the intestine varies considerably. The ascending colon has the largest number of bacteria, followed by the terminal ileum (10^11^/g vs. 10^7^–10^8^/g), compared with no more than 10^2^–10^3^/g in the proximal ileum and jejunum. The host provides a suitable environment and sufficient nutrients for the intestinal microbiota, which in turn participates in the regulation of multiple functions of the body, such as affecting the growth and metabolism of the host, regulating the immune system, inhibiting the growth of pathogens, and maintaining the integrity of the intestinal barrier and the balance of the internal environment.^3^ In the process of shaping the intestinal microbiota, dietary changes can account for 57% of the differences in the intestinal microbiota, compared with 12% caused by gene changes, suggesting that diet plays a leading role in the formation of the intestinal microbiota.^4^ Western diets, which tend to be high-sugar and high-fat, can lead to metabolic disorders affecting the host gastrointestinal metabolism and immune balance.^5^ A protein-rich diet increases the activity of bacterial enzymes, such as β-glucuronidase, azo reductase, and nitroreductase, and produces toxic metabolites, which leads to inflammation.^6^ Therefore, diet is one of the most important environmental factors regulating the composition and function of the intestinal microbiota.

ARGs are increasingly prevalent in the environment, and have been found in water^7^ and food supply chains.^8^ Increasing amounts of ARGs such as *Klebsiella pneumoniae* carbapenemase (KPC-1) and New Delhi metallo-β-lactamase (NDM-1)-producing bacteria have been associated with multidrug resistance.^9^ Additionally, *mcr-1*-positive Enterobacteriaceae (MCRPE) have been found in food and environments in over 50 countries across four continents, and *mcr*-like elements have been detected in a variety of plasmid types including IncI2, IncHI2, and IncX4.^10^ More worrisome is extensive horizontal transfer of *KPC-1,NDM-1* and *mcr-1* in various Gram-negative bacteria, especially *Escherichia coli* and *Klebsiella pneumoniae*, which is accelerating the spreading of ARGs and badly compromising the treatment of infections, resulting in substantial morbidity and mortality.^11^ In addition, these ARGs can easily enter intestines along with water and food,^12^ where resident organisms can serve as potential receptor bacteria, and the appropriate temperature and rich nutrients in intestines are conducive to the reproduction and diffusion of ARB.^13^ Many studies have shown that there are many types of ARGs in intestines, and they can be transferred between intestinal microbiota through plasmids.^14^

One study found that horizontal gene transfer (HGT) is common in the human intestinal microbiota and occurs by various mechanisms, the most typical of which are transduction and conjugation transfer.^15^ Through conjugation transfer, many species in the same habitat can obtain ARGs, which are usually transferred across species boundaries.^16^ The high density of bacteria in the intestine can facilitate the transfer of plasmids between the same species or between different species.^17^ The high diversity of ARGs in human bacteria may lead to antibiotic resistance of human pathogens in the future. Once ARGs enter human pathogens, they may be transmitted to symbiotic bacteria through pathogens, then further spread among symbiotic bacteria, causing greater harm.^18^ In 2017, the World Health Organization published the first list of “priority pathogens” possessing antibiotic resistance.^19^ The list consists of 11 species, including *Staphylococcus aureus, Helicobacter pylori*, and *Salmonella typhi*. Eight of these can participate in DNA uptake through natural ability, and the three *Enterobacteriaceae* on the list potentially have this ability.^20^ Conjugation-mediated plasmid transfer may be the main reason to promote the emergence of highly pathogenic or antibiotic resistant pathogens.^21^ The main reason why they are considered represent a great threat to human health is that they can not only cause serious diseases, but are also resistant to currently effective antibiotics.

In addition to the risk of entering pathogenic bacteria, ARGs may also enter intestinal symbiotic bacteria, and ARGs may lie hidden in the intestinal microbiota for a long time. Even short-term use of antibiotics can leave ARGs in the human intestinal tract for several years.^22^ Some types of intestinal microbiota may be selected as receptor bacteria for further transmission after exogenous ARGs enter the intestine, and existing technology cannot successfully eradicate them.

The diet can not only provide energy for intestinal microbiota, it can also remodel them. However, there are few reports on whether and how diet affects the amplification and transfer of ARGs in the intestinal microbiota. The present study explored the effect of diet on the dissemination profiles of exogenous ARGs in the intestinal tract by analyzing the spatial and temporal characteristics of the colonization and transfer of ARGs, changes in the intestinal microbiota, membrane permeability, reactive oxygen species (ROS), the distribution of receptor bacteria, the types and quantities of intestinal resistance groups, and the correlation between ARGs and intestinal microbiota. The findings may help to provide a new approach for controlling the spread of ARGs.

## 2. Results

### 2.1. Diet alters the composition and metabolites of the intestinal microbiota in mice

**Figure 1.**
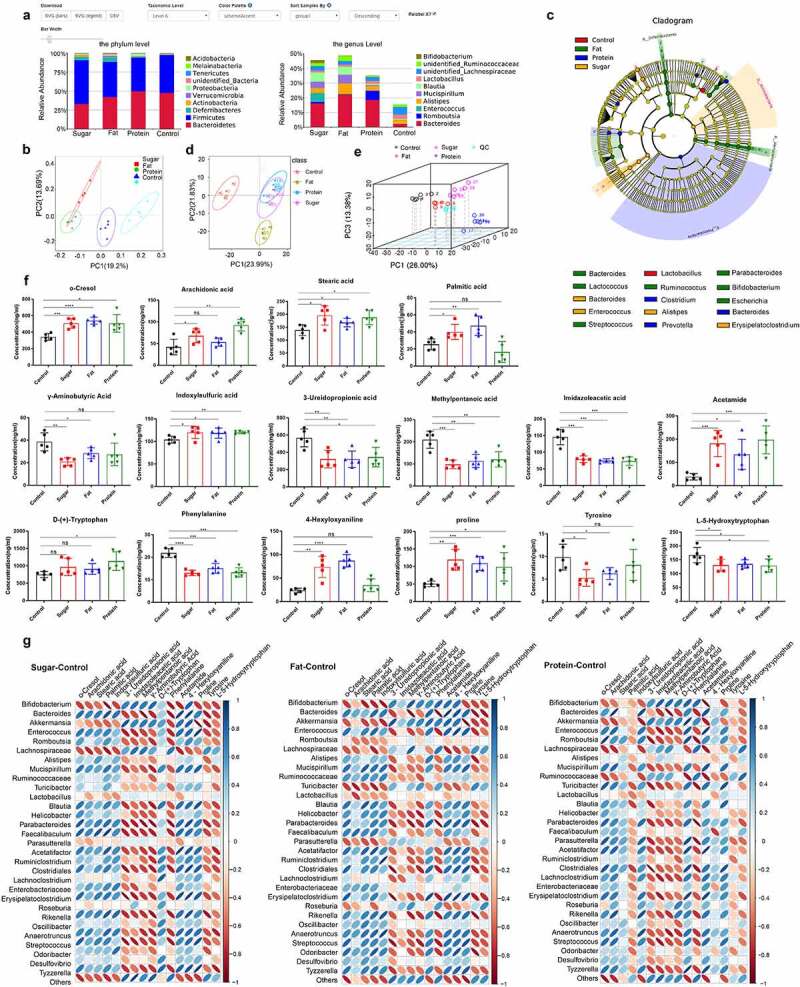
The intestinal microbiota and its metabolites shaped by different diets. The relative abundance of species at the phylum and genus levels (excluding others) of the four dietary groups (a). The intestinal microbiota of the four groups was analyzed by unweighted UniFrac distance principal coordinate analysis (PCoA) (b). In the LEfSe cladogram, the inner to outer radiating circles represent the taxonomic level from phylum to genus, and the diameter of small circles is proportional to the relative abundance. Species with no significant differences are uniformly colored yellow, and species with differences are colored with the biomarker group (c). Principal component analysis (PCA) of metabolomics data for the four dietary groups (d) and multivariate PCA of the top three principal components (e). Differential metabolites related to inflammation in each diet group (f). Thermogram analysis of the correlation between different bacteria and different metabolites; Blue indicates a positive correlation and red indicates a negative correlation (g).

High-sugar, high-fat, and high-protein diets could significantly alter the structure of the intestinal microbiota, and each diet had its own distribution patterns (Figure 1a). At the phylum level, *Bacteroidetes* was most dominant in the control group (51.55%), followed by the high-protein group (51.19%), the high-fat group (41.17%), and the high-sugar group (33.96%). *Bacteroidetes* and *Firmicutes* accounted for more than 96.98% in the control group, 93.03% in the high-protein group, 89.66% in the high-fat group, and 86.3% in the high-sugar group. In addition, *Actinobacteria* were more abundant in the high-sugar group (5.27%), while *Verrucomicrobia* were abundant in the high-fat group (2.90%), and *Proteobacteria* were abundant in the high-protein group (2.94%). At the genus level, in the high-sugar group, *Bacteroides* (16.00%) was the most abundant, followed by *Blautia* (5.03%), *Enterococcus* (4.87%), *Mucispirillum* (4.30%), *Akkermansia* (4.16%), and *Alistipes* (3.70%). In the high-fat group, *Bacteroides* (21.89%) was the most abundant, followed by *Alistipes* (6.61%), *Mucispirillum* (5.52%), *Blautia* (5.13%), and *Akkermansia* (3.21%). In the high-protein group, *Bacteroides* (16.94%) was dominant, followed by *Romboutsia* (7.59%), *Alistipes* (2.92%), *Lactobacillus* (1.64%), and *Mucispirillum* (1.18%). In the control group, the most abundant genus was *Lactobacillus* (2.76%), followed by *Alistipes* (2.33%) and *Bacteroides* (1.99%).

Principal coordinate analysis (PCoA) based on weighted UniFrac distance showed that some samples of high-sugar and high-fat diet communities coincided, and high-sugar, high-fat, and high-protein diet all diverged from the normal diet (Figure 1b). Based on LEfSe analysis, there were significant differences in bacteria among groups (bacterial biomarkers). The biomarkers were *Enterococcus, Bacteroides, Lactococcus, Alistipes, Blautia, Erysipelatoclostridium*, and *Lachnoclostridium* in the high-sugar group, but were *Akkermansia, Parabacteroides, Lactococcus, Ruminococcus, Bifidobacterium, Escherichia, Helicobacter*, and *Streptococcus* in the high-fat group. The high-protein group included *Desulfovibrio, Clostridium, Bacteroides, Prevotella, Turicibacter*, and *Marvinbryantia*. Finally, *Parabacteroides, Ruminiclostridium, Lactobacillus, Eubacterium*, and *Lachnospiraceae* were the markers in the normal group (Figure 1c). These results showed that the intestinal microbiota was significantly remolded by high-sugar, high-fat, and high-protein diets, compared with the normal diet, which indicates that a new intestinal microbiota may form to adapt to the dietary pattern.

Principal component analysis (PCoA) of metabolites revealed significant differences between the metabolites of high-sugar, high-fat, and high-protein diets and the control group (Figure 1d-e, Table 1). Analysis of specific metabolites showed that there were significant differences in some lipids, amino acids, and fatty acids, and many metabolites were associated with inflammation pathways. Furthermore, we assessed metabolites that may damage the intestinal mucosal barrier and cause inflammation, such as o-cresol, stearic acid, palmitic acid, indoxylsulfuric acid, acetamide, and 4-hexyloxyanilane, and proline, which were significantly higher in the high-sugar and high-fat groups than in the control group (*p* < .05). Compared with the control group, except for palmitic acid and 4-hexyloxyanilane (*p* > .05), the other metabolites associated with inflammation in the high-protein group were at significantly higher levels (*p* < .05). In addition, short chain fatty acids (SCFAs) including methylpentanoic acid, imidazoleacetic acid, 3-ureldopropionic acid, and γ-aminobutyric acid were detected, and SCFAs in the high-sugar and high-fat groups were significantly lower than in the control group (*p* < .05). Except for γ-aminobutyric acid (*p* > .05), other SCFAs in the high-protein group were significantly less abundant than those in the control group (*p* < .05). In addition, several amino acids were analyzed, and levels of L-5-hydroxytryptophan and phenylalanine in the high-sugar, high-fat, and high-protein groups were significantly lower than those in the control group (*p* < .05). Tyrosine in the high-sugar and high-fat groups was significantly lower than in the control group (*p* < .05), and there was no significant difference compared with the high-protein group (Figure 1f).

**Table 1.** Metabolite results from PERMANOVA tests

Group	Df	Sums of squares	Mean squares	F Model	Variation (R^2^)	Pr
Control/Fat	1	0.23758669	0.237587	8.297696	0.509133	0.004
Control/Sugar	1	0.177125864	0.177126	7.343092	0.478593	0.006
Control/Protein	1	0.177520284	0.17752	5.619834	0.412621	0.008
Fat/Sugar	1	0.11402714	0.114027	6.691307	0.45546	0.008
Fat/Protein	1	0.122816775	0.122817	5.011328	0.385151	0.009
Sugar/Protein	1	0.090140785	0.090141	4.507848	0.360402	0.017

Df, degree of freedom.

R^2^, Contribution of a group to differences (the larger the R^2^, the greater the contribution).

Pr, *p* < .05 (high reliability).

Correlation analysis between bacteria with significant differences at the genus level and differential metabolites after comparing the high-sugar, high-fat, and high-protein groups with the control group respectively. The results showed that, in high-sugar group, *Bacteroides, Enterococcus, Helicobacter, Ruminiclostridium*, and *Desulfovibrio* were positively correlated with metabolites such as o-cresol, stearic acid, indoxylsulfuric acid, proline, and tryptophan, but were negatively correlated with SCFAs such as acetic acid, butyric acid, and propionic acid. High-fat and high-protein groups had similar correlation compared with the control group, and there was only difference in the strength of the correlation (Figure 1g).

### 2.2. Diet affects the temporospatial distribution of ARG amplification and transfer in the intestine

**Figure 2.**
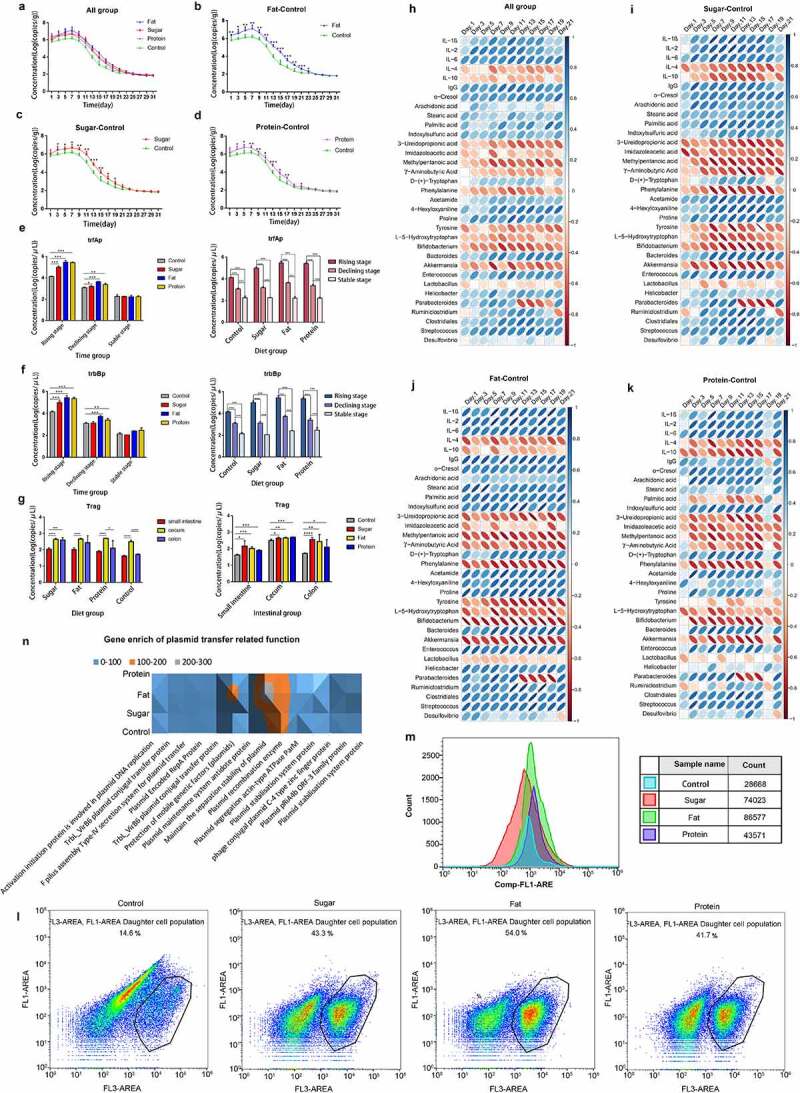
Distribution and mechanisms of ARGs in different diets. Temporal distribution of ARGs in different diet groups (a); 1–7 days is the rising stage, and ARB were administered by gavage every day at this stage; 9–21 days is the declining stage, and the stable stage began 23 days later. The figure shows the difference in time distribution between each of the three diet groups and the control group (b–d). Expression levels of ARG conjugation transfer regulatory genes (e, f). The distributions are shown for two regulatory genes promoting the conjugation and transfer of ARGs in the three stages for the four dietary groups. The spatial distributions of ARGs in different dietary groups are shown for the small intestine, cecum, and colon (g). Pearson correlation analysis of ARG amplification and transfer (distribution level at each time point) with inflammation-related indicators (serum immune factors and inflammation-related metabolites) for all diet groups (h) and for high-sugar, high-fat, and high-protein groups shown as a heatmap. Blue indicates a positive correlation and red indicates a negative correlation, and the darker the color, the stronger the correlation (i–k). Scatter plot of cell membrane-damaged bacteria bound to PI dye (l). Fluorescence intensity of bacteria combined with DCFH-DA (m).The total amount of gene enrichment in plasmid transfer-related pathways for each diet group, the abundance is represented by gray, Orange and blue from high to low (n).

The temporal distributions of exogenous ARG amplification and transfer were monitored after long-term consumption of each diet. The diets were fed to remodel the intestinal microbiota of mice, and during ARB exposure for 7 days, the content of target ARGs in feces was continuously monitored. The results of real-time PCR showed that the patterns of all diets could be divided into three stages: a rising stage from 1–7 days, a declining stage from 8–23 days, and a stable stage after 25 days. All groups reached the peak value of 10^6^ copies/g on the 7th day, then entered the declining stage after gavage, and continued to decline to ~10^2^ copies/g, and maintained this level thereafter (Figure 2a). Comparing the differences between the three special diets and the control group revealed that the high-fat diet had a strong promoting effect on the amplification and transfer of ARGs, reaching 10^7^ copies/g at the peak. From the first day to the 23rd day, amplification and transfer of ARGs was significantly higher in the high-fat diet group than the control group (*p* < .05), and there was no significant difference after the 23rd day (*p* > .05; Figure 2b). Levels of ARGs in the high-sugar group were significantly higher than in the control group from the 3rd day to the 19th day (*p* < .05), and there was no significant difference after the 19th day (*p* > .05) (Figure 2c). Amplification and transfer of ARGs in the high-protein group were significantly higher than in the control group from the 3rd day to the 17th day and the 21st day (*p* < .05), but there were no significant differences or other time points (*p* > .05) (Figure 2d). The results showed that high-sugar, high-fat, and high-protein diets could promote the amplification and transfer of exogenous ARGs in the intestine. The high-fat diet had the strongest and most long-lasting promoting effect, followed by the high-sugar diet and the high-protein diet.

We analyzed the expression of *trfAp* and *trbBp* during the three stages (rising, declining, and stable) under different diets. According to detection of the *Trag* gene in the fecal genome, we selected one time point for each of the three stages to measure expression of *trfAp* and *trbBp*. The results showed that in each diet group, expression of the genes in the rising stage was significantly higher than in the declining and stable phases (*p* < .001), and expression in the declining stage was also significantly higher than in the stable stage (*p* < .001). Expression of *trfAp* in the high-fat, high-sugar, and high-protein groups was significantly higher than that in the control group (*p* < .001). In the stable stage, there were no significant differences between groups (*p* > .05) (Figure 2e). Expression of *trbBp* in each diet group was similar to that of *trfAp*. In the rising stage, expression in the high-fat, high-sugar, and high-protein groups was significantly higher than in the control group (*p* < .001). In the declining stage, the high-fat and high-protein groups exhibited significantly higher expression levels than the control group (*p* < .01), but there was no significant difference between the high-sugar and control groups (*p* > .05). In the stable stage, there were no significant differences between the groups (*p* > .05) (Figure 2f). The results suggest that the effects of different diets on the conjugation and transfer of ARGs may be due to the regulation of gene expression, thereby promoting the conjugation and transfer of ARGs.

In order to further explore the spatial distribution of the above-mentioned exogenous ARGs in the gut, the intestinal tract was dissected when the ARGs in the feces of mice were stable, and the content of ARGs in each intestinal segment was determined by fluorescence quantitative PCR. The results showed that ARGs in the colon and cecum in the high-sugar group were significantly higher than in the small intestine (*p* < .001). ARGs in the cecum in the high-fat group were significantly more abundant than in the small intestine (*p* < .0001), and levels in the colon were higher than in the small intestine, but the difference was not significant (*p* > .05). ARGs in the cecum in the high-protein group were significantly higher than in the small intestine (*p* < .0001), and the colon (*p* < .05). ARGs in the cecum in the control group were significantly more abundant than in both the small intestine and the colon (*p* < .0001). In addition, ARGs in the small intestine were significantly higher in the high-sugar, high-fat, and high-protein groups than in the control group (*p* < .05). In the cecum, ARGs in high-sugar and high-fat groups were significantly higher than in the control group (*p* < .05), and the difference between high-protein and control groups was more significant (*p* < .01). In the colon, ARGs in high-sugar, high-fat, and high-protein groups were significantly higher than in the control group (*p* < .05; Figure 2g). The results showed that high-sugar, high-fat, and high-protein diets could promote the amplification and transfer of exogenous ARGs in all intestinal segments, among them, the cecum may be the main site for the amplification and transfer of ARGs.

Pearson correlation analysis of high-sugar, high-fat, and high-protein groups showed that levels of exogenous ARGs were significantly correlated with inflammatory markers, including serum immune factors, inflammatory-related metabolites, and inflammatory-related bacteria. Among them, pro-inflammatory factors, including interleukin 1β (IL-1β), IL-2, IL-6, and immunoglobulin G (IgG) and pro-inflammatory metabolites (o-cresol, indoxysulfuric acid, stearic acid, proline, etc.) and bacteria that destroy the intestinal mucosal barrier (*Enterococcus, Clostridiales, Streptococcus*, etc.) were positively correlated with levels of exogenous AGRs. The high-fat group had the highest positive correlation, followed by high-sugar and high-protein groups. Additionally, anti-inflammatory factors (IL-4, IL-10) and anti-inflammatory metabolites (methylpentanoic acid, imidazoleacetic acid, 3-ureldopropionic acid, γ-aminobutyric acid, phenylalanine), and bacteria that protect the intestinal mucosal barrier (*Bifidobacterium, Akkermansia*, and *Lactobacter*) were negatively correlated with levels of exogenous AGRs. The high-sugar group had the highest positive correlation, followed by high-fat and high-protein groups (Figure 2h-k). This suggests that inflammation may be related to the amplification and transfer of ARGs in the gut.

In order to further explore changes in bacterial structure and function occurring under a diet-induced inflammation microenvironment, we measured the cell membrane permeability of intestinal microbiota in each group by flow cytometry. Bacteria with damaged cell membranes in the normal diet group accounted for 14.6% of total bacteria, compared with 43.3% in the high-sugar group, 54.0% in the high-fat group, and 41.7% in the high-protein group (Figure 2l). Thus, high-fat, high-sugar, and high-protein diets can destroy the cell membrane and increase its permeability to a certain extent, which promotes plasmid conjugation and transfer. In addition, ROS produced in bacterial cells induced by diet can directly react with biomolecules, affecting cellular processes. The results showed that high-sugar, high-fat, and high-protein diets could significantly increase intracellular ROS. Compared with the control group, ROS levels in the high-sugar, high-fat and high-protein groups were 2.58-fold, 3.02-fold, and 1.52-fold higher, respectively (Figure 2m). The above results showed that the inflammatory microenvironment generated by high-fat, high-sugar, and high-protein diets can effectively stimulate bacteria to produce moderate levels of ROS and trigger the bacterial SOS response, thereby promoting plasmid conjugation and transfer. Finally, analysis of certain functional pathways related to plasmid conjugation and transfer showed that compared with the normal diet control group, more genes were enriched in these pathways in high-sugar, high-fat, and high-protein groups (Figure 2n). Among them, the high-fat group contained the most enriched genes, followed by high-sugar and high-protein groups. Therefore, the correlation between diet-induced inflammation and the transfer of ARGs may be reflected by the above aspects.

### 2.3. Diet affects the distribution of ARGs receptor bacteria

**Figure 3.**
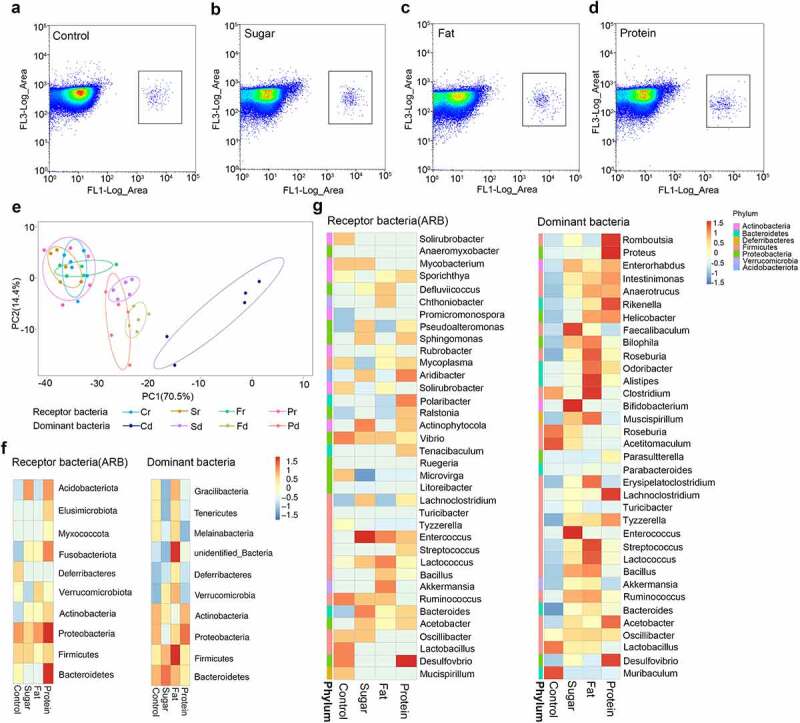
The distribution of receptor species in different diet groups. Flow cytometry was used to select receptor bacteria with green fluorescence, and positive bacteria were collected in a small square frame (a–d). PCA of receptor bacteria (diet-r) and dominant bacteria (diet-d) for the four dietary groups analyzed by unweighted UniFrac distance (e). Top 10 species distribution heatmap at the phylum level for receptor bacteria and dominant bacteria in each diet group (f). Top 35 species distribution heatmap at the genus level for receptor bacteria and dominant bacteria in each diet group, different colors on the left of the heatmap indicate the phylum to which each genus belongs (g).

In order to identify receptor bacteria, mouse feces were collected 7 days after feeding donor bacteria, when the conjugation of ARGs with intestinal microbiota reached the maximum. Bacteria were sorted by flow cytometry for species identification. The results of flow cytometry showed that receptor bacteria (microbiota receiving exogenous ARGs) could be distinguished from the intestinal microbiota and gathered in a certain area (Figure 3a-d). The results of bacterial PCA showed that receptor bacteria for the four diets were clustered in a certain area, and there were large differences in the high-protein group. In each diet group, there is a certain distance between the distribution of receptor bacteria and dominant bacteria, with the high-sugar, high-fat, and high-protein groups closer, and the normal diet group further away. This shows that the dominant bacteria shaped by high-sugar, high-fat, and high-protein diets are more similar to their receptor bacteria than for the normal diet. In other words, the dominant bacteria shaped by high-sugar, high-fat, and high-protein diets are more likely to accept ARGs as receptor bacteria than those of the normal diet (Figure 3e).

Analysis of receptor bacteria at the phylum level showed that the receptor bacteria in each diet group mainly belonged to *Proteobacteria, Firmicutes, Bacteroidetes, Actinobacteria, Acidobacteriata, Verrucomicrobia, Fusobacteriota, Deferibacters*, and *Myxococcota*. Among them, *Proteobacteria* accounted for 58.68% in the high-sugar group, 53.43% in the high-fat group, 60.12% in the high-protein group, and 47.15% in the normal diet group (Figure 3f). These results suggest that *Proteobacteria* may be more likely to become a suitable host for RP4 plasmids carrying exogenous ARGs. Further analysis of receptor bacteria showed that those in the high-sugar diet group were mainly distributed in *Lachnoclostridium, Mycobacterium, Enterococcus, Pseudoalleromonas, Sphingomonas, Aridibacter, Actinophytocola, Bacteroides*, and *Vibrio*. In the high-fat diet group, receptor bacteria were mainly distributed in *Defluviicoccus, Chthoniobacter, Enterococcus,Lactococcus, Bacillus, Akkermansia*, and *Ruminococ-cus*. In the high-protein diet group, receptor bacteria were mainly distributed in *Sporichthya, Vibrio, Enterococcus, Sphingomonas, Sphingomonas, Polaribacter, Tenacibaculum, Bacteroides*, and *Desulfovibrio*. In the normal diet group, receptor bacteria were mainly distributed in *Solirubrobacter, Mycobacterium, Vibrio, Ruminococcus, Lactobacillus*, and *Desulfovibrio* (Figure 3g).

Compared with the dominant bacteria in the corresponding diet at the phylum level (top 10) and the genus level (top 35), some receptor bacteria present in high abundance in each diet group were the same as the dominant bacteria, indicating that some receptor bacteria came from the dominant bacteria. Bacteria in high-sugar, high-fat, and high-protein groups overlapping at the phylum level included *Proteobacteria, Firmicutes*, and *Actinobacteria*, while bacteria in the normal diet group overlapping at the phylum level were *Proteobacteria, Firmicutes*, and *Deferribcteres*. At the genus level, *Lachnoclostridium, Enterococcus, Lactococcus, Ruminococcus, Bacteroides*, and *Acetobacter* were common in the high-sugar group, while *Akkermansia, Ruminococcus, Bacillus, Lactococcus*, and *Ruminococcus* were common in the high-fat group, *Lachnoclostridium, Desulfovibrio, Acetobacter*, and *Streptococcus* were common in the high-protein group, and *Lactobacillus, Desulfovibrio, Oscillibacter*, and *Mucispirillum* were common in the normal diet group. In addition, the top 10 bacteria were counted at the phylum level, of which six species of donor bacteria and receptor bacteria were the same, accounting for 94% of total bacteria. The top 35 bacteria were counted at the genus level, of which 15 species of donor bacteria and recipient bacteria were the same, accounting for 68% of total bacteria. This shows that the dominant bacteria of each diet group are the main target for transfer of ARGs in this environment. This indicates that the dominant bacteria in the intestinal microbiota shaped by different diets can also become potential receptor bacteria. When donor and receptor bacteria are present in the same habitat, exogenous ARGs tend to be transferred not only to *Proteobacteria*, but also to the dominant bacteria in the different environment. And among these dominant bacteria, there are some probiotics (*Lactobacillus, Bifidobacterium*) and pathogenic bacteria (*Streptococcus, Vibrio*). This suggests that different diets play a guiding role in the selection of receptor bacteria by exogenous ARGs.

### 2.4. Diet affects the resistance genome of the intestinal microbiota

**Figure 4.**
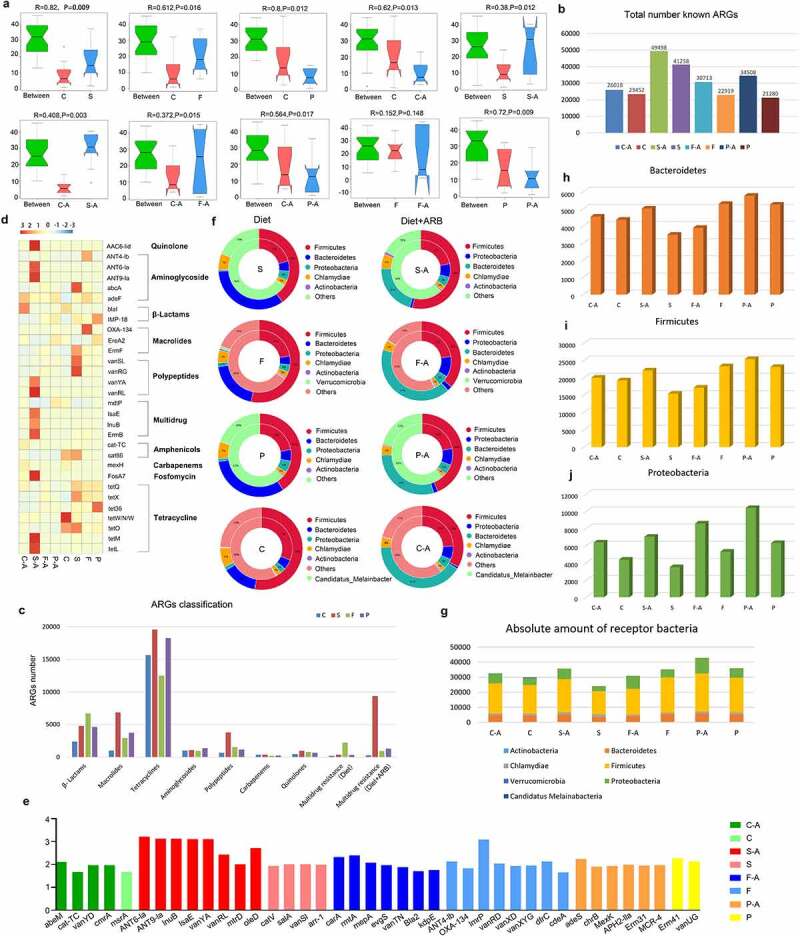
Intestinal resistance gene annotation in different diet groups. Anosim analysis based on the abundance of ARGs in each diet group with and without added ARB (a). Histogram of the total number of ARGs in each group (b). Classification histogram of ARGs in each group (c). Cluster heatmap of the distribution and relative abundance of ARGs in each group (d). Histogram of ARG markers before and after adding ARB in each diet group (e). Double circle diagram of the proportion of ARGs distributed among species. The inner circle is the distribution of species containing ARGs, and the outer circle is the distribution of species with all genes (f). Absolute number of different bacteria containing ARGs in each group (g–i).

In order to analyze differences between diet groups, anosim analysis was conducted based on the abundance of ARGs. The results showed that there were significant differences between the high-sugar (S, *p* = .009), high-fat (F, *p* = .016), and high-protein (P, *p* = .012) groups and the normal diet group (C), indicating that different diets affected the intestinal resistance genome. Among them, the effect of the high-sugar diet was the most significant. There were significant differences between the high-sugar add ARB group (S-A, *p* = .003), the high-fat add ARB group (F-A, *p* = .015), the high-protein add ARB group (P-A, *p* = .017), and the normal diet add ARB group (C-A). Comparing the same diet with and without ARB revealed significant differences between the normal diet add ARB group (C-A) and the normal diet group (C; *p* = .013), the high-sugar diet add ARB group (S-A) and the high-sugar diet group (S; *p* = .012), and the high-protein diet add ARB group (P-A) and the high-protein diet group (P; *p* = .009). However, there was no significant difference between the high-fat diet add ARB group (P-A) and the high-fat diet group (P; *p* = .148; Figure 4a). Thus, based on different dietary effects, the addition of exogenous ARB may affect the intestinal resistance genome, with distinct characteristics for different treatments. Among them, the number of intestinal resistance genes increased significantly when exogenous ARB were added to the high-sugar, high-fat, and high-protein groups (Figure 4b). Classification of resistance genes showed that in the control group, tetracycline resistance genes were the most abundant, followed by β-lactam resistance genes. In the high-sugar group, tetracycline resistance genes were the most abundant, followed by macrolides and β-lactam resistance genes. In the high-fat group, tetracycline resistance genes were the most abundant, followed by β-lactam genes, and this was the same for the high-protein group. The abundance of multidrug resistance (MDR) genes was ordered high-sugar group, high-fat group, high-protein group, and control group, from high to low. After adding ARB, MDR genes were significantly increased in the S-A group and P-A group (Figure 4c).

In order to analyze the distribution of antibiotic resistance ontology (ARO) in each group, the top 30 ARO genes were selected to draw an abundance cluster heatmap (Figure 4d). The results showed that the ARO genes with the highest abundance in the high-sugar group were *abcA, ErmF, vanSL, vanRG, cat86, tetX*, and *tetO. AAC6-lid, ANT6-la, ANT9-la, vanYA, vanRL, lsaE, lnuB, ErmB, FosA7, tetM*, and *tetL* were the most abundant ARO genes in the high-sugar plus ARB group. The ARO genes with the highest abundance in the high-fat group were *ANT4-lb, adeF, OXA-134, ErmF, tetQ*, and *tetX. TetQ, vanRG*, and *adeF* were the most abundant ARO genes in the high-fat plus ARB group. The ARO genes with the highest abundance in the high-protein group were *IMP-18, EreA2, tetQ, tetX*, and *tet36. AdeF, IMP-18, EreA2*, and *mdtP* were the most abundant ARO genes in the high-protein plus ARB group. The ARO genes with the highest abundance in the normal diet group were *blal, cat86, tetO*, and *tetW/N/W. AdeF, blal, cat-TC, mexH*, and *tetQ* were the most abundant ARO genes in the normal diet adding ARB group.

The distribution of the above ARGs mainly included aminoglycosides, β-lactams, peptides, amphenicols, multidrug resistance genes, and tetracyclines. In addition, ARG markers were identified by absolute abundance analysis of ARGs in each diet group, revealing a high abundance of *tetQ, adeF, tetW/N/W* (absolute abundance >1000) and *ErmF, tetX, tetO*, and *cat86* (absolute abundance between 1000 and 100) in each diet group before and after adding ARB. These results indicate that these resistance genes could be abundant in the intestinal microbiota in different diets with or without exogenous ARB, and were not affected by external factors. In addition, there were specific ARGs in different diets before and after adding exogenous ARB, such as *abeM, cat-TC, vanYD*, and *cmrA* in the normal diet add ARB group (C-A). In the normal diet group (C), gene marker was *msrA*. Gene markers in the high-sugar diet add ARB group (S-A) were *ANT6-Ia, ANT9-Ia, lnuB, lsaE, vanYA, vanRL, mtrD*, and *oleD*. Gene markers in the high-sugar diet group (S) were *catV, salA, vanSI* and *arr-1*. Gene markers in the high-fat diet add ARB group (F-A) were *carA, mepA, rmtA, evgS, vanTN, Bla2* and *kdpE*. In high-fat diet group (F), gene markers were *ANT4-Ib, OXA-134, lmrP, vanRD, vanXD, vanXYG, dfrC, EBR-1-β-lactamase*, and *cdeA*. Gene markers *adeS, chrB, MexK, APH2-IIa, Erm31*, and *MCR-4* were identified in the high-protein diet add ARB group (P-A). Gene markers in the high-protein diet group (P) were *Erm41* and *vanUG* (Figure 4e). The above results showed that different diets can affect the distribution of ARGs, and the abundance of some specific ARGs can increase following consumption of different diets, implying that diet can shape the intestinal antibiotic resistance genome. Addition of exogenous ARB can further shape the intestinal antibiotic resistant genome following consumption of different diets.

Using the double circle diagram of the relationship between resistance genes and species, we further analyzed the species in which resistance genes were distributed in each group (Figure 4f). The results showed that ARO genes were mainly distributed in *Firmicutes, Bacteroidetes, Proteobacteria, Chlamydiae*, and *Actinobacteria* in the high-sugar group, and after adding ARB, *Proteobacteria* increased from 5% to 7%. ARO genes were mainly distributed in *Firmicutes, Bacteroidetes, Proteobacteria, Chlamydiae, Actinobacteria*, and *Verrucomicrob-ia* in the high-Fat group, and after adding ARB, *Proteobacteria* increased from 4% to 11%. In the high-protein group, ARO genes were mainly distributed in *Firmicutes, Proteobacteria, Bacteroi-detes, Chlamydiae* and *Actinobacteria*, and *Proteobacteria* increased from 6% to 9% after adding ARB. In the normal diet group, ARO genes were mainly distributed in *Firmicutes, Bacteroidetes, Proteobacteria, Chlamydiae, Actinobacteria*, and *Candida melinabacter*, and *Proteobacteria* increased from 3% to 9% after adding ARB. By counting the absolute number of these bacterial phyla that distribute ARGs, we found that although the number of *Firmicutes* was the highest, changes before and after ARB addition in each diet group were not consistent, and likewise for *Bacteroidetes* (Figure 4g-4i). *Proteobacteria* was significantly higher in all ARB-containing diet groups (C-A, S-A, F-A, P-A) than in non-ARB diet groups (C, S, F, P), and the abundance of *Proteobacteria* in the high-sugar, high-fat, and high-protein groups was significantly higher than in the normal diet group, among which the abundance of *Proteobacteria* in the high-protein group was the highest (Figure 4j).

## 2.5. Correlations between intestinal ARGs and bacteria in different diet groups

**Figure 5.**
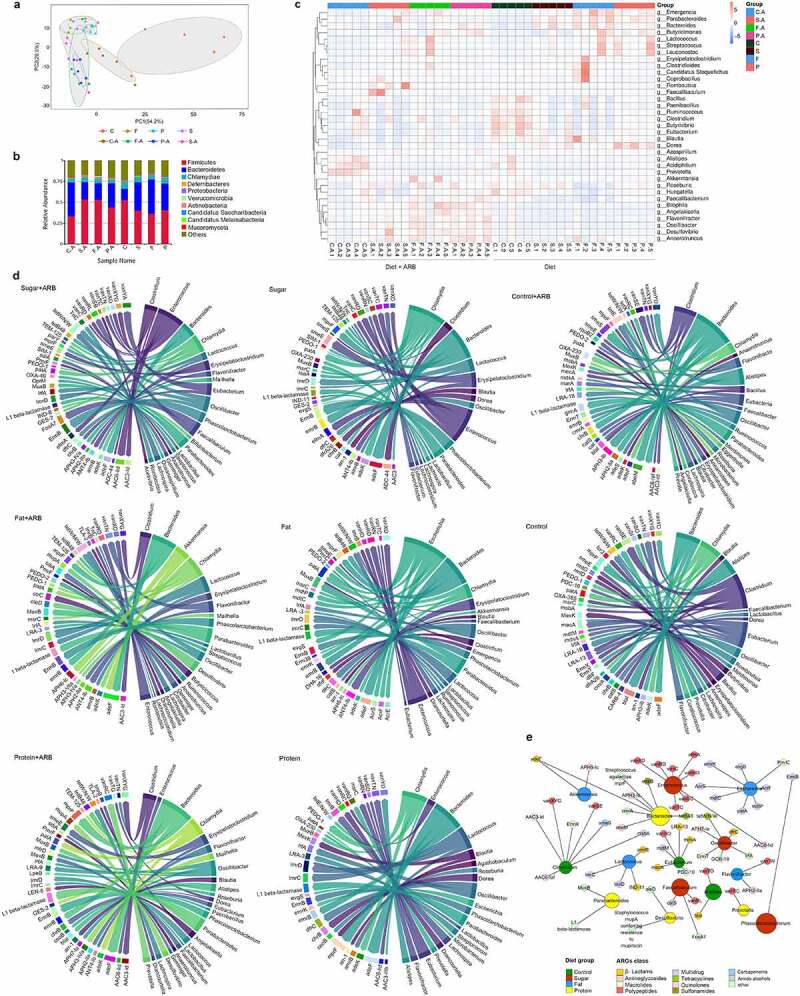
The relationship between resistance genes and corresponding species. PCA analysis of the intestinal microbiota in each diet group before and after adding ARB (a). Histogram showing the distribution of bacteria in each diet group before and after adding ARB is shown at the phylum level (b). The distribution of ARGs and a cluster heatmap of the relative abundance of samples in different diet groups before and after adding ARB (c). Overview of the relationship between ARGs and species in different diet groups; ARGs are on the left, and species are on the right (d). Network of the relationship between high-abundance ARGs and the distribution of bacteria in each diet group (e).

After adding ARB to each diet, the intestinal microbiota in high-sugar (*p* = .004), high-fat (*p* = .012), and high-protein (*p* = .008) groups were clustered together, and there were significant differences compared with the normal diet control group. The high-sugar add ARB group (*p* = .007), high-fat add ARB group (*p* = .012), and high-protein add ARB group (*p* = .041) were clustered together, and there were significant differences from the normal diet group. There was a significant difference between the high-fat add ARB group and the high-fat group (*p* = .028). There was a significant difference between the high-protein add ARB group and the high protein group (*p* = .008), but there was no significant difference between the high-sugar add ARB group and the high-sugar group (*p* = .083; Figure 5a). Therefore, the effect of diet on the intestinal microbiota was significantly greater than that of exogenous bacteria. In addition, there were significant differences in the distribution of the intestinal microbiota at the phylum level among diet groups before and after adding ARB (Figure 5b). Further analysis of the distribution of the top 30 bacteria at the genus level before and after adding ARB revealed a high abundance of *Bacteroides, Ruminococcus, Clostridium, Eubacterium*, and *Alistipes* in the normal diet group and the normal diet add ARB group. There was a high abundance of *Bacteroides, Erysipelatoclostridium, Clostridium, Faecalibaculum*, and *Roseburia* in the high-sugar group and the high-sugar add ARBgroup. *Bacteroides, Erysipelatoclostridium, Clostridium, Akkermansia*, and *Parabacteroides* were abundant in the high-fat group and the high-fat add ARB group. *Bacteroides, Parabacteroides, Butyricimonas, Erysipelatoclostridium, Dorea*, and *Lactococcus* were abundant in the high-protein group and the high-protein add ARB group (Figure 5c).

By analyzing the number of ARGs in different bacteria in each diet group, we found that *Bacteroides, Alistipes, Parabacterioides, Clostridium, Chlamydia, Oscillibacter*, and *Eubacter* possessed more ARGs in the C-A group. In the S-A group, *Phascolarctobacterium, Enterococcus, Faecalibaculum, Eubacterium, Bacteroides, Oscillibacter, Clostridium, Lachnospira*, and *Chlamydia* had more ARGs. In the F-A group, *Bacteroides, Akkermansia, Phascolarctob-acterium, Parabacteroides, Lactococcus, Flavonifract-or, Chlamydia*, and *Erysipelatoclostridium* harbored more ARGs. In the P-A group, *Bacteroides, Parabacteroides, Clostridium, Prevotella, Chlamydia, Erysipelatoclostridium*, and *Phascolarctobacterium* included more ARGs. In the C group, *Alistipes, Clostridium, Bacteroides, Chlamydia, Eubacterium, Flavonifractor*, and *Merdimonas* had more ARGs. In the S group, *Bacteroides, Phascolarctobacterium, Erysipelatoclostridium, Enterococcus, Chlamydia, Lactococcus*, and *Clostridium* possessed more ARGs. In the F group, *Bacteroides, Parabacteroides, Erysipelatoclostridium, Escherichia, Enterococcus, Bacillus*, and *Phascolarctobacterium* harbored more ARGs. In the P group, *Bacteroides, Lactococcus, Chlamydia, Bacillus, Parabacteroides, Erysipelatoclos-tridium and Dorea* included more ARGs. In conclusion, *Bacteroide, Clostridium, Erysipelatoclostr-idium*, and *Enterococcus* possessed more ARGs (Figure 5d).

Specific analysis of the distribution of ARGs in the corresponding bacteria in each group showed that *Bacteroides* had the most types and the highest number of ARGs. Therefore, symbiotic bacteria such as *Bacteroides*, which account for a large proportion of the intestinal microbiota, are more likely to become MDR bacteria. Some bacteria of *Firmicutes* are similar, such as *Enterococcus* and *Lactococcus*. They are abundant in the gut, hence they have a greater chance of receiving ARGs and becoming ARB. Some pathogenic bacteria were also identified, such as *Chlamydia* and *Clostridium*. Although *Chlamydia* contained the most different types of ARGs, the abundance of ARGs was not highest in these organisms. This indicates that the number of ARGs depends on the proportion of host bacteria in the intestine to a certain extent. In addition, *Alistipes, Clostridium*, and *Eubacterium* in group C and C-A, *Phascolarctobacterium, Enterococcus*, and *Faecalibacum* in group S and S-A, *Akkermansia, Clostridium*, and *Lactococcus* in group F and F-A, and *Parabacteroides, Dorea*, and *Prevotella* in group P and P-A had a high abundance of ARGs. This indicates that the dominant bacteria in different diets could be the main target for ARG colonization. Additionally, bacteria with a high abundance of ARGs were significantly increased after ARB addition to each diet group. Thus, invasion of exogenous ARB can disrupt the distribution of the original bacteria, allowing more members of the intestinal microbiota to accept ARGs and become new ARB. When considering groups before and after addition of ARB as a single group, *Enterococcus, Faecalibaculum*, and *Phascolarctobacterium* contained high abundance of ARGs in the high-sugar diet, *Akkermansia, Escherichia, Flavonifractor*, and *Lactococcus* contained a high abundance of ARGs in the high-fat diet, and *Bacteroides, Parabcteria, Desulfovibrio*, and *Prevotella* contained a high abundance of ARGs in the high-protein diet. In the normal diet, *Clostridium, Alistipes*, and *Eubacterium* contained a high abundance of ARGs (Figure 5e). These results suggest that bacteria remodeled by diet may be the main target of most ARGs in the gut.

These bacteria include intestinal symbiotic bacteria such as *Bacteroides, Enterococcus, Ruminoco-ccus, Prevotella, Parabacterioides, Eubacterium*, and *Faecalibaculum*, which can promote digestion and metabolism. Some can produce butyric acid, acetic acid, formic acid, and other beneficial SCFAs through fermentation. However, some are pathogenic. For example, *Clostridium* and *Chlamydia* can cause a variety of diseases, *Escherichia* can cause severe diarrhea, and *Erysipelatoclostridium* can cause severe infection. These results showed that when resistance genes select receptor bacteria, although they may be selective toward closely related bacteria or the dominant bacteria in the environment, symbiotic bacteria and pathogenic bacteria might not be distinguished. Because they are in the same niche, they may accept ARGs and become ARB, thereby inhibiting antibiotic treatment and representing a serious threat to human health.

## 3. Discussion

This study found that under the effect of various diets, the period during which exogenous ARGs enter the intestine can be divided into three stages: the rising stage, the declining stage, and the stable stage. In the rising stage, ARGs can reach ~10^6^ copies/g; in the declining stage, levels drop continuously from 10^6^ copies/g to 10^2^ copies/g; in the stable stage, the content remains stable at ~10^2^ copies/g. During the experiment, when the supply of exogenous ARB was stopped, exogenous ARGs gradually decreases to ~10^2^ copies/g. Consistent with our observation, exogenous ARGs can proliferate and disseminate to indigenous gut microbiota via HGT, but the quantity of transconjugants in the gut declined to a lower level in the declining stage,^13^ which may be attributed to the fitness cost of plasmids. Although plasmids provide bacteria with new adaptive tools, they also entail a metabolic burden that, in the absence of selection for plasmid-encoded traits, reduces the competitiveness of the plasmid-carrying clone.^23^ Furthermore, there may be plasmid incompatibility between plasmids. The same negative feedback mechanism is used for the same types of plasmids. The more plasmids, the stronger the inhibition of replication, the slower the replication, and eventually many bacteria lose plasmids.^24^ This is why plasmids only exist in small numbers in the intestinal microbiota. However, when bacteria are placed under antibiotic selection pressure, plasmids can be quickly mobilized to replicate, and transferred to other bacteria through horizontal or vertical gene transfer to resist the threat of antibiotics. This may also be a strategy evolved by the intestinal microbiota to balance the contradiction between fitness cost and antibiotic pressure.

In addition, high-sugar, high-fat, and high-protein diets promote the amplification and transfer of ARGs in the intestine, which may be related to the inflammatory microenvironment caused by these three diets (Supplementary file S2). Evidence from experimental models suggests that although the intestinal microbiota usually drives immune activation, chronic inflammation in turn shapes the intestinal microbiota and leads to dysbacteriosis.^25^ High-sugar, high-fat, and high-protein diets can further activate the immune responses of regulatory T cells^26^ by affecting the intestinal microbiota and its metabolites,^27^ or stimulating the synthesis and secretion of toxic H_2_S, cresol, indole, and ammonia, which can increase intestinal permeability, damage intestinal cell DNA, interrupt intestinal cell metabolism and growth, and eventually lead to intestinal inflammation.^28^ Inflammation triggers the bacterial SOS response by producing many stressors^29^ (such as ROS), and promoting horizontal transfer of ARGs and the redistribution of virulence factors.^17^ Among these responses, moderate ROS production is conducive to increasing the frequency of junction transfer.^30^ In addition, high-sugar, high-fat, and high-protein diets may cause intestinal microbiota imbalance through inflammation, resulting in a decrease in colonization resistance of symbiotic bacteria,^31^ while the chances of donor and opportunistic bacteria engaging will increase significantly, thereby increasing the rate of ARG transfer. Under normal conditions, HGT between *Enterobacteriaceae* is blocked by symbiotic microbiota, mainly by maintaining the total density of facultative anaerobic bacteria at a fairly low level, ≪10^8^ colony-forming units (CFU)/g,^32^ through colonization resistance of symbiotic microbiota. Following intestinal inflammation caused by diet, a transient outbreak of intestinal bacteria can occur,^33^ characterized by reduced diversity and increased abundance of some bacteria, causing a rapid acceleration in recombination and gene transfer. These outbreaks may facilitate the redistribution of plasmid-encoded genes between pathogens and symbionts, thereby increasing the suitability of plasmid-encoding and the transmission of determinants of ARGs.^34^ Additionally, environments with low diversity, with a simple microbiota composition but high abundance of specific taxa, are more conducive to the amplification and transfer of ARGs. Thus, high-sugar, high-fat, and high-protein diets may promote the amplification and transfer of ARGs through the interaction between the induced microbiota and its metabolites and the intestinal inflammatory microenvironment. Among them, the high-fat group showed the most severe inflammation, and therefore promoted the greatest amplification and transfer of ARGs, followed by the high-sugar and high-protein groups. This process can occur in all segments of the intestine, but mainly in the cecum and colon. The cecum is located between the small intestine and colon, which is the thickest, shortest, and most metabolically active part of the whole gut. The cecal bacterial concentration can be as high as 10^11^–10^12^ CFU/mL, hence it provides a base for receptor bacteria and a favorable place for bacterial conjugation and transfer. Because the small intestine is close to the stomach and the pH value is low, it is not conducive to the survival of bacteria, hence the bacterial content can be as low as 10^3^–10^4^ CFU/mL,^35^ and few receptor bacteria are available for conjugation and transfer. The amount of ARGs detected in different intestinal segments differs, mainly due to the intestinal structure and differences in the types and amounts of bacteria.

High-sugar, high-fat, and high-protein diets can significantly promote the amplification and transfer of ARGs. Furthermore, high-fat, high-sugar, and high-protein diets can promote the expression of regulatory genes *trfAp* and *trbBp*, which can stimulate the conjugation and transfer of ARGs. The *trfAp* gene encodes the initiation protein of plasmid replication, which can stabilize plasmid replication in many bacterial species.^36^ Also, *trfAp* is involved in the transmembrane transport and conjugation of plasmid DNA under the regulation of many operons. Conjugation requires cell-cell contact and the formation of conjugation pairs under the action of related regulatory genes.^30^ The protein encoded by the *trbBp* gene is responsible for processing and modifying the fimbriae on the cell surface, enabling cells to connect and build fimbriae protein channels, forming an efficient pairing system, and providing favorable conditions for DNA replication and transfer in the next step.^37^ Diet-induced inflammation led to changes in metabolic pathways in the intestinal microbiota, accompanied by changes in some gene functions. We analyzed several functions related to plasmid conjugation and transfer, including activating initiation protein (through similarity) involved in plasmid DNA replication, TrbL_Virb6 plasmid-binding transfer protein, the F pili assembly type IV secretion system for plasmid transfer, and plasmid-encoded RepA protein. We found that more genes were enriched in the high-fat, high-sugar, and high-protein groups than in the control group. This shows that in these diet groups, relatively more regulatory genes were mobilized to play a role in pathways related to plasmid conjugation and transfer, and thereby promote the transfer of ARGs. Therefore, increased expression of regulatory genes promoted by diet may induce the amplification and transfer of ARGs.

Following exogenous ARB carrying ARGs entering the intestine, the main receptor bacteria belong to *Proteobacteria*. In addition, we chose the RP4 plasmid carried by ARB, and directly administered it by gavage. The results showed that most of the RP4 plasmid was also enriched in *Proteobacteria* (Supplementary File S3). It is reported that carbapenems and extended spectrum agents work well against opportunistic *E. coli* and *K. pneumoniae*, while β-lactamase resistance genes are easily transmitted to *Proteobacteria* in the gut.^38^ In complex populations, even without selection pressure, distantly related bacteria can receive broad host range plasmids from donor bacteria.^39^ When broad host range plasmids (RP4) entered into complex communities, a core super-permissive fraction of the bacterial community can acquire plasmid from the donor strain, and *Proteobacteria* was the main hosts for broad host range plasmids,^13,40,41^ consistent with our results. It seems that *Proteobacteria* members are more likely to accept plasmids and maintain them in the latter stages. This may be because *Proteobacteria* strains have a unique ability to achieve efficient contact between cells through specific mating-mediating pheromones.^42^ In addition, analysis of the plasmid genomes and microbiome involvement of *Proteobacteria* revealed a large and highly diverse plasmid-encoded helper gene library, which is conducive to efficient binding and HGT,^43^ increasing the probability of plasmid uptake and conjugative transfer. Gene exchange is most frequent among bacteria of the same phylum, and most HGT (60%) occurs between closely related members of the same phylum.^41^ In this process, interactions between donor bacteria, receptor bacteria, and plasmids affect the transfer efficiency.^44^ Yano et al.^45^ reported that genetic differences between closely related IncP-1 plasmids through plasmid skeleton evolution can lead to significant differences in host range efficiency without affecting their broad host range properties. In addition, comparative analysis of plasmid sequences shows that the evolutionary host range of IncP-1a(RP4) plasmids seems to be mainly limited to *Proteobacteria*,^46^ and the fitness factors encoded by this plasmid are conducive to the growth of zygotes of *Proteobacteria* and the efficiency of the conjugation process.^47^

In our study, some high-abundance bacteria shaped by diet are also important hosts of ARGs. Examples include *Enterococcus, Lactococcus, Ruminococcus, Bacteroides, Acetobacter* in the high-sugar group. *Akkermansia, Ruminococcus, Bacillus*, and *Lactococcus* in the high-fat group; *Lachnoclostridium, Desulfovibrio, Acetobacter*, and *Streptococcus* in the high-protein group; and *Lactobacillus, Desulfovibrio, Oscillibacter*, and *Mucispirillum* in the normal diet group. Studies have shown that the same types of ARGs in different bacteria can be detected in a variety of environments, and their preference depends on the environmental niche.^48^ This shows that when bacteria in a specific environment share the same habitat with ARB, they have a greater chance of receiving drug-resistant genes and become new ARB. Therefore, ARGs are not random but selective in their host distribution. They may be preferentially shared between bacteria that are closely related, or transferred from bacteria strongly adapted to the same niche as a new host, which may be more conducive to transfer and expression at a lower fitness cost. Herein, different diet shaped own core super-permissive microbiota that could acquire ARGs from donor bacteria, and the microbiota comprised 68% of identified transconjungants.

With the development of metagenomics technology, we can not only determine the species and abundance of bacteria, but also analyze the distribution of ARGs in the whole intestinal microbiota resistance genome. The results of intestinal resistance genome analysis revealed more than 4 × 10^6^ ARGs, and more than 20,000 known ARGs in the intestinal microbiota of the four diet groups. This shows that antibiotic abuse leads to the ubiquitous distribution of ARGs, and even specific pathogen-free (SPF) mice that have not been exposed to antibiotics can contain many ARGs, inherited from their mother or grandmother.^49^ The abundance of ARGs was analyzed, and genes related to tetracycline were the most abundant. Tetracycline is a broad-spectrum antibiotic that has been widely used since it was discovered in the 1940s. However, as tetracycline is widely used as a growth promoter and added to animal feed, the tolerance of bacteria to tetracycline is becoming stronger and stronger; in some areas, 66.9% of *E. coli* and 44.9% of *Klebsiella* are tetracycline resistant.^50^These results indicate that ARGs contaminating the environment may eventually accumulate in the intestines of animals. Different diets can further regulate the distribution of ARGs on this basis, and the high-sugar, high-fat, and high-protein groups contained significantly more ARGs than the control group. By comparing the distribution of ARGs in species before and after adding ARB, it was found that *Proteobacteria* increased significantly in each diet containing ARB group than in the corresponding ABR-free diet, suggesting that exogenous ARGs entered *Proteobacteria* easily. In addition, each group had a high abundance of *Bacteroides*, and *Bacteroides* contained the most different types and the highest abundance of ARGs. There are reports of ARGs in *Bacteroides* in different microecological environments.^48^ In addition, there were specific ARG markers in each diet group, and most were distributed among bacterial markers shaped by each diet, further proving that some dominant bacteria shaped by each diet can become important hosts of ARGs, demonstrating that the transfer of ARGs has a certain selectivity.

Although transfer of ARGs displays a certain selectivity between species, it seems that this selectivity does not include selection between symbiotic bacteria and pathogenic bacteria. In our current study, we found that the distribution of ARGs in these bacteria includes intestinal symbiotic bacteria, such as *Bacteroides, Enterococcus, Ruminococcus, Prevotella, Parabacterioides, Eubacterium, Faecalibacum*, and others. They not only promote digestion and metabolism, but also endow the host with multiple functions that promote immune homeostasis and immune responses, and prevent pathogen colonization.^51^ These functions include producing butyric acid, acetic acid, formic acid, and other beneficial SCFAs through fermentation. This may be beneficial for the colonization of symbiotic bacteria when ARGs enter symbiotic bacteria, by keeping the structure and abundance of the bacteria stable in the face of antibiotics and exogenous bacteria. However, it is also possible that they are transferred to other pathogenic bacteria. Some studies have reported that *Lactobacillus* carry ARGs and are resistant to antibiotics.^51^ Some may even transfer their inherent ARGs to other pathogens through HGT,^52^ thereby threatening human health. In addition, some ARGs are directly distributed in pathogenic bacteria, such as *Clostridium* and *Chlamydia*, which can cause a variety of diseases, and *Escherichia* and *Erysipelatoclostridium*, which are common in severe infections.^53^ Once the infection is rampant, it places a great burden on the host, and is difficult to treat due to the protective effects of ARGs. During clinical treatment, bacteremia patients infected with carbapenem-resistant *Enterobacter-iaceae* or carbapenem-resistant *Pseudomonas aeruginosa* suffer 40–70% mortality.^54^ Therefore, when selecting receptor bacteria, ARGs exhibit selectivity for bacteria that are close relatives and/or the dominant bacteria in the environment. However, symbiotic and pathogenic bacteria may not be distinguished because they occupy the same niche and may accept ARGs to become ARB, which may compromise the effects of antibiotic treatment and endanger host health. There is a potential risk associated with diets containing excessive nutrients such as high-sugar, high-fat, and high-protein diets. Therefore, when choosing diets, priority should be given to diets that protect the intestinal microbiota and intestinal health, such as those rich in dietary fiber.

In conclusion, the intestinal inflammatory microenvironment remolded by high-sugar, high-fat, and high-protein diets may be conducive to the spreading of ARGs, which mainly occurs in the cecum. Transfer of ARGs displays a certain selectivity for specific receptor bacteria, and tends to involve transfer to the dominant bacteria in a given dietary environment. Furthermore, depending on diet, exogenous ARGs can also alter the metagenomics and resistance genome of the intestines, representing a serious health threat.

## 4. Materials and Methods

### 4.1. Construction of fluorescent donor bacteria

The donor bacterium (MEC-5) isolated from mouse feces was identified as *Escherichia coli*. Donor strains were chromosomally tagged with *mCherry* encoding constitutive red fluorescence and the *tac* promoter expressing *mCherry* was encoded upstream.^55^ In addition, RP4 plasmids harboring tetracycline (*tet*) and kanamycin (*km*) resistance genes were appended to a genetically encoded expressible green fluorescent protein (GFP) gene. Next, MEC-5-mCherry was electroporated with the plasmid RP4-GFP-Tet^R^Km^R^. As a result, both red and green fluorescence occurs in donor cells, but upon plasmid transfer to a fecal bacterium, the transconjugants display green fluorescence due to GFP expression, which can be detected and sorted by fluorescence microscopy or fluorescent-activated cell sorting (Supplementary File S1).

### 4.2. Animal experiments

Eight-week-old male SPF Balb/c mice (Beijing Weitonglihua Experimental Animal Company, Beijing, China) were fed for 1 week. Fresh feces were collected and antibiotic resistance of the intestinal microbiota was assessed using antibiotic-sensitive paper (Beijing SanYao Science and Technology Development Company, Beijing, China). The target plasmid was detected by agarose gel electrophoresis. Mice were divided into four groups: high-sugar diet (60%), high-fat diet (60 kcal%), high-protein diet (40%), and normal diet^56^ (Beijing Xiaoshuyoutai Biological Company, Beijing, China). After mice were fed different diets for 8 weeks to stabilize the inflammatory state, mice in each diet group whose intestinal microbiota had changed significantly were divided into experimental (gavaged with ARB) and blank control (gavaged with normal saline) groups. Mice in the ARB group were gavaged with 200 μL of bacterial solution (10^9^ CFU/mL) once a day for 1 week. Mice in each experimental group (Plasmid groups) were gavaged with 200 μL of plasmid solution at a cell density of 10^9^ CFU/mL, while mice in the control group were gavaged with the same amount of normal saline. After centrifugation at 3300 × g for 5 min, bacterial cells cultured overnight at 37°C were resuspended in normal saline, and the cell density was adjusted to 10^9^ CFU per 200 μL. Mice were fed and watered freely, weighed once a week, and feces were collected and frozen for storage.

### 4.3. Quantitative PCR (qPCR) detection of target genes

Genomic DNA from fecal and intestinal samples were extracted using a Fecal Genome Extraction Kit (Qiagen, Dusseldorf, Germany). Primers were designed according to the *Trag* fragment of the RP4 plasmid-specific gene.^13^ A standard curve was drawn using plasmid standards, and target genes were detected by fluorescence qPCR. Total RNA was extracted from feces using a Total RNA Extraction Kit (Qiagen). RNA was reverse-transcribed into cDNA by a reverse transcription kit (Bao Bioengineering Company, Dalian, China), and fluorescence qPCR was performed to detect regulatory genes.

### 4.4. Separation of receptor bacteria by flow cytometry

Fecal suspensions were placed in an ultrasonic crusher for ~20 min before being passed through 70 μm and 10 μm filter screens. The bacterial suspensions were diluted to 10^5^–10^6^ cells with phosphate-buffered saline (PBS). Samples were analyzed in a flow cytometer (Bio-Rad ZE5, California, USA) equipped with a 488 nm laser. The green fluorescence was collected in the FITC channel (530 ± 15 nm). The red fluorescence was collected in the PE channel (585 ± 20 nm). After separating all bacterial liquids, samples were placed in centrifuge tubes containing 100 μL PBS (pH = 7.0) to collect green fluorescent bacteria for subsequent sequencing. To explore the mechanisms through which diet stimulates HGT, flow cytometry was employed to differentiate and quantify diet-exposed and control cells. Fluorescence intensity is a function of cell membrane permeability, with higher fluorescence intensity signifying enhanced cell membrane permeability. A 1 mL sample of bacterial suspension was stained with 10 μL of propidium iodide (PI, 1 mg/mL; OMEGA, Georgia, USA) and incubated in the dark for 8 min before measurement. The concentration of the bacterial suspension was always <10^6^ cells/mL. The fluorescence intensity was measured at 488–525 nm.^57^ The reagent 2ʹ,7ʹ-dichlorofluorescein diacetate (DCFH-DA; Invitrogen, California, USA) was used to quantify intracellular ROS generation. Specifically, bacterial suspensions (10^6^–10^7^ CFU/mL) were stained by DCFH-DA (at a final concentration of 10 μM) for 20 min at 37°C in darkness. Bacterial suspensions were then washed by PBS, and the fluorescence intensity was measured at 488–525 nm to determine the generation of ROS by bacteria.^30^

### 4.5. Sequencing the intestinal microbiota

After genomic DNA was extracted from fecal samples using the cetyltrimethylammonium bromide method, the purity and concentration of DNA were determined and PCR amplification was performed. PCR products were analyzed by agarose gel electrophoresis and target bands were recovered. A DNA PCR Sample Preparation Kit (Illumina Truseq, San Diego, California, USA) was used to construct the library. After quantification by qubit (Thermo Fisher, Waltham, Massachusetts, USA) and qPCR, the quality of the library was determined, and the library was sequenced on a Novaseq 6000 Illumina system (Illumina Truseq). The original data were spliced with flash (v1.2.11, http://ccb.jhu.edu/software/FLASH/)^58^ to generate original tags, and clean tags were obtained by strictly filtering the raw data. Qiime (V2.0, http://qiime2.org/^59^) was employed for quality control. Tag sequences were compared with the species annotation database, and chimeric sequences were removed to generate the final valid tags (https://github.com/torognes/vsearch/). Uparse software (v7.0.100, http://www.drive5. com/uparse/) was used to cluster all valid tags of all samples (97%). The Mothur method in SILVA138 (http://www.arb-silva.de/)^60^ and the SSUrRNA database (threshold 0.8–1)^61^ was used to obtain taxonomic information for each taxonomic level and to determine the number of species in each sample community. Finally, data for each sample were normalized, and samples with the smallest amount of data were taken as the standard for normalization. Finally, α-diversity analysis and β-diversity were assessed based on normalized data.

### 4.6. Determination of intestinal metabolites

After tissue samples were ground in liquid nitrogen, 500 μL of 80% methanol water solution containing 0.1% formic acid was added. After vortex oscillation, samples were incubated in an ice bath for 5 min, then centrifuged at 15,000 × *g* and 4°C for 10 min. Part of each supernatant was diluted with mass spectrometry (MS)-grade water until the methanol content reached 53%, centrifuged in a centrifuge tube at 15,000 × *g* and 4°C for 10 min, and analyzed by liquid chromatography MS (LC-MS).^62^ Blank samples comprised 53% methanol water solution containing 0.1% formic acid, and the pretreatment process was the same as for treatment samples. The data file was imported into Compound Discoverer 3.1 (Thermo Fisher, Massachusetts, USA) for simple screening of retention time, mass charge ratio, and other parameters, and peaks in different samples were aligned according to a retention time deviation of 0.2 min and a mass deviation of 5 ppm to make the identification more accurate. The peak area was quantified, target ions were integrated, the molecular formula was predicted based on molecular ion peak and fragment ions, and compared with mzCloud (https://www.mzcloud.org/). Background ions were removed using data from blank samples, and quantitative results were normalized. Finally, data identification was achieved and quantitative results were obtained.

### 4.7. Metagenomics determination of the intestinal resistance genome

The concentration and purity of the fecal genome were determined using Qubit4 (Thermo, Massachus-etts, USA). Readfq (V8, https://github.com/cjfields/readfq) was used to preprocess the original data from the sequencing platform, and the preprocessed data were assembled and analyzed by SOAPdenovo software.^63^ The scaftigs generated by single sample and mixed assembly containing fragments <500 bp^64^ were filtered out. Metagenemark (V2.10, http://topaz.gatech.edu/GeneMark/) was used to predict the open reading frames (ORFs) of samples and scaftigs (≥500 bp),^65^ and fragments <100 nt were filtered out. Bowtie2 (Bowtie2.2.4) was used to compare the effective data for each sample with the initial gene catalog, and the number of reads in each sample was calculated. Genes with ≤2 reads in each sample were filtered out^66^ and the final gene catalog for subsequent analysis was obtained. Based on the number of reads and the gene length, the abundance of each gene in each sample was calculated. Based on the abundance of each gene in the gene catalog for each sample, basic statistics were derived. The resistance gene identifier software associated with the CARD database (https://card.mcmaster.ca/) was used to compare unigenes with the CARD database (RGI built-in blastp, default value ≤1e-30).^67^ According to the comparison of RGI values and the abundance of unigenes, antibiotic resistance ontology (ARO) was determined and analyzed.

### 4.8. Statistical analysis

GraphPad Prism V7.0 (https://www.graphpad.com/) and RStudio (https://www.rstudio.com/) were used for statistical analysis and mapping. Body weight, fecal excretion, and immune factors of mice in each group were analyzed by unpaired t-tests, Dunnett’s multiple comparison tests, or one-way analysis of variance (ANOVA) of Tukey’s multiple comparison tests. Statistical significance was set at 0.05, hence *p* > .05 means no statistical significance. The significance of pairwise comparisons is indicated by asterisks (*p < .05, **p < .01, ***p< .001). In α-diversity analysis, qiime software (v2.0, http://qiime2.org/) was used to calculate Chao1 and Shannon indices. Difference analysis of the α-diversity index between groups was conducted with parametric and nonparametric tests. Since the experiment involved more than two groups, Tukey and Wilcox tests were performed. For β-diversity, the ggplot2 (V4.05) program in the R software package (https://www.r-project.org/) was used to draw a principal coordinate analysis (PCoA) diagram, heatmap, and overview circle diagram. R software was used to analyze differences between groups for β-diversity, and parametric and nonparametric tests were carried out. The Anosim, MRPP, and Adonis functions of the R vegan package were also used.

## Supplementary Material

Supplemental Material

## Data Availability

All data used to support the results of this study, including macrogenome data (bioproject ID: prjna753725) and 16S sequencing data (bioproject ID: prjna718065), have been uploaded to NCBI.
