# Supplementary material for: High-sugar, high-fat, and high-protein diets promote antibiotic resistance gene spreading in the mouse intestinal microbiota
Source: Gut Microbes. 2022 Jan 14;14(1):2022442. doi: 10.1080/19490976.2021.2022442 (PMC8765071; doi:10.1080/19490976.2021.2022442)
Supplement: Supplemental Material [file KGMI_A_2022442_SM9719.zip › supplementary/Supplementary File.docx]

**1 Construction and identification of double fluorescent labeled donor bacteria**

At the beginning of this study, we first cultured fresh fecal bacterial suspensions of mice using *Escherichia coli*-selective medium (Difco m Endo Agar LES, BD), and selected pure strains according to the color and morphology of colonies. After purifying the isolated strain many times, it was identified as *E. coli* by OptiRead (Thermo, Massachusetts, USA) ^1^ based on 32 biochemical indices (**Supplementary Table 1**). OptiRead further assessed the antibiotic resistance of the strain toward 26 common antibiotics using antibiotic sensitivity plates, and detected sensitivity to some antibiotics, including tetracycline and kanamycin^2^ (**Supplementary Table 2**).Secondly, the strain was identified as *E. coli* by matrix-assisted laser desorption/ionization time-of-flight mass spectrometry (Shimadzu-Biotech Corp., Kyoto, Japan) ^3^. The whole genome of bacteria was sequenced by NovaSeq PE150, the relevant coding genes were retrieved by the GeneMarkS program, and the strain was sequenced. The above detection and sequence alignment analyses confirmed that the strain was *E. coli* ^4^ (**Supplemental Fig.1a,b**).Therefore, *E. coli* (EC-5) was obtained from mouse intestinal microbiota. The whole-genome sequencing results included 89 antibiotic resistance genes (ARGs) based on the CARD database (**Supplementary Table 3**). We modified the identified *E. coli* genome by incorporating the *mCherry* gene, obtained E. coli (MEC-5) expressing *mCherry* (emitting red fluorescence) (**Supplemental Fig.1c-h**), introduced the RP4 plasmid containing the tetracycline resistance gene (Tet), the kanamycin resistance gene (Km), and expressing green fluorescent protein (GFP) as a donor strain (**Supplemental Fig.1i,j**).

**
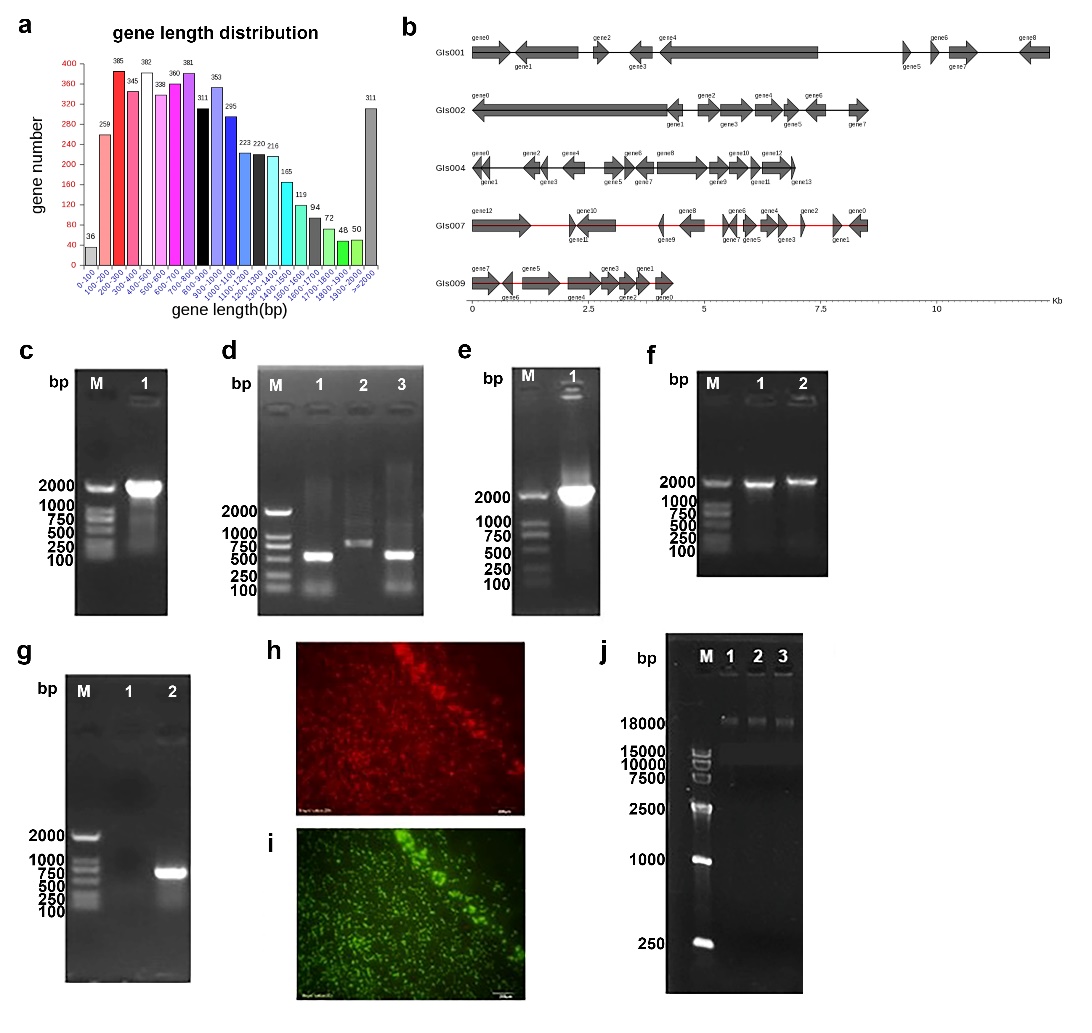
**

**Identification of bacteria and construction of double fluorescently-labelled donor bacteria.** Gene length distribution map of the sample (**a**). The abscissa is the gene length, and the ordinate is the number of corresponding genes. The abscissa of the statistical map of gene distribution in the sample gene island is the length scale (the gene Island shown in the figure is less than 15 kb in length) (**b**). Products amplified by MEC-5 msbB-UP-F/MEC-5 msbB-down-R (**c)** and amplified msbB upstream (UP) homologous arm fragments, *mCherry* fragments, and msbB downstream (down) homologous arm fragment products (**d**), and Mec-5 msbB up *mCherry* msbB down repair homologous fusion fragments (**e**) detected by agarose gel electrophoresis. After insertion of MEC-5 msbB UP-F/E5-msbB-down-R into *mCherry*, positive clones were screened. The newly constructed MEC-5 and wild-type strain DNA correspond to the amplified products (**f**). Agarose gel electrophoresis of *mCherry*-F/*mCherry*-R products (**g**). *mCherry* was inserted into MEC-5 (*E. coli*) and red fluorescence was detected by fluorescence microscopy (**h**). The RP4-GFP-Tet^R^Km^R^ plasmid was electrotransformed into MEC-5-*mCherry* for green fluorescence detection (**i**) and agarose gel electrophoresis analysis (**j**).

**Supplemental Table 1 EC-5** **biochemical reaction identification results**

| **Biochemical index** | **Reaction type** | **Biochemical index** | **Reaction type** |
| --- | --- | --- | --- |
| FR1 | - | Uric acid | - |
| Xylose | + | FR12 | - |
| FR3 | - | Trehalose | + |
| Malt dust | + | FR4 | - |
| FR5 | - | Fructose | + |
| Arabinose | + | Lysine | + |
| FR7 | + | Arginine | - |
| Malonic acid | - | Pyruvic acid | + |
| Ornithine | - | Sorbitol | + |
| Sucrose | - | FR9 | + |
| FR8 | - | Mannitol | + |
| Inositol | - | FR10 | + |
| Aescin | - | Arabinol | - |
| TDA | - | Raffinose | - |
| FR6 | + | Cellobiose | - |
| Citrate | - | Guanidine butyric acid，Herring spermine | - |

**Supplemental Table 2 EC-5 antibiotic susceptibility results**

| **Antibiotic** | **Tolerance concentration (μg/mL)** | **Judgment result** |
| --- | --- | --- |
| Gemifloxacin | ≤ 0.015 | I |
| Kanamycin | ≤ 1 | S |
| Polymixin B | ≤ 0.5 | I |
| Cefepime | ≤ 0.25 | R |
| Doxycycline | ≤ 0.12 | S |
| Levofloxacin | 2 | R |
| Aztreonam | ≤ 1 | R |
| Minocycline | 2 | R |
| Colistin | 1 | I |
| Meropenem | ≤ 0.06 | R |
| Streptomycin | 8 | I |
| Amikacin | ≤ 4 | I |
| Doripenem | ≤ 0.12 | S |
| Ertapenem | ≤ 0.25 | S |
| Tigecycline | ≤ 0.25 | I |
| Ceftazidime | ≤ 1 | R |
| Cefotaxime | 1 | R |
| Gentamicin | ≤ 1 | I |
| Ciprofloxacin | ≤1 | R |
| Tetracycline | ≤ 0.5 | S |
| Cefazolin | 4 | I |
| Cefoxitin | ≤ 2 | S |
| Ampicillin | ≤ 2 | S |
| Chloramphenicol | ≤ 2 | S |
| Sulfisoxazole | ≤ 32 | I |
| Nalidixic Acid | ≤ 2 | I |

S: sensitivity, R: resistance, I: intermediary.

**Supplemental Table 3 Resistance genes contained in EC-5**

| **ARO name** | **Bit score** | **ARO name** | **Bit score** | **ARO name** | **Bit score** |
| --- | --- | --- | --- | --- | --- |
| acrA | 167.548 | gadX | 486.878 | MuxB | 390.578 |
| AcrS | 447.973 | golS | 115.546 | NmcR | 162.925 |
| adeL | 149.058 | hmrM | 411.764 | novA | 248.054 |
| arlR | 117.857 | H-NS | 127.872 | oleC | 148.673 |
| arlS | 127.102 | hp1181 | 139.813 | OprM | 145.976 |
| arnA | 135.961 | kdpE | 134.42 | patA | 131.724 |
| bacA | 543.117 | LamB | 907.131 | patB | 163.31 |
| baeR | 486.493 | leuO | 610.527 | PmrC | 1052.35 |
| baeS | 128.257 | lmrD | 137.117 | PmrE | 792.341 |
| basS | 150.214 | macA | 192.971 | PmrF | 662.914 |
| bcr-1 | 135.961 | macB | 120.553 | rob | 124.79 |
| bcrA | 135.191 | marA | 111.309 | rosA | 516.924 |
| chrB | 139.043 | MCR-3 | 157.147 | rosB | 162.925 |
| clbB | 147.132 | MdfA | 313.153 | rphB | 189.889 |
| cmeB | 170.244 | mdtA | 127.102 | sav1866 | 144.05 |
| cmlv | 148.288 | mdtE | 129.028 | sdiA | 369.777 |
| CMY-63 | 310.842 | mdtF | 1459.12 | smeR | 126.331 |
| cpxA | 129.028 | mdtG | 330.487 | sul3 | 124.79 |
| CpxR | 136.732 | mdtH | 801.201 | TaeA | 127.872 |
| CRP | 434.491 | MdtK | 647.506 | tcmA | 162.54 |
| dfrA3 | 127.487 | mdtL | 124.405 | TolC | 996.882 |
| dfrE | 206.068 | mdtN | 142.895 | vanG | 148.673 |
| emrA | 791.186 | mdtO | 1385.16 | vanHD | 189.119 |
| emrD | 749.969 | mdtP | 979.548 | vanRB | 113.62 |
| emrE | 105.916 | mecC | 154.836 | vanRE | 114.39 |
| emrK | 152.14 | MexA | 146.362 | vanRI | 120.939 |
| emrY | 129.413 | mexN | 362.844 | vanTC | 144.436 |
| evgA | 417.157 | mfd | 240.35 | vanTE | 152.91 |
| evgS | 189.119 | msbA | 158.688 | YojI | 1106.66 |
| gadW | 137.117 | msrB | 291.197 |  |  |

**2 Inflammation of mice in each diet group**

After feeding different diets for 8 weeks, the systemic immunity and intestinal immunity of mice was altered by varying degrees. Analysis of serum cytokines showed that levels of proinflammatory factors IL-1, IL-β, IL-2, IL-6, and IgG in high-sugar, high-fat, and high-protein groups were significantly higher than those in the control group, and anti-inflammatory factors IL-4 and IL-10 were significantly lower than in the control group. In addition, the intestinal permeability marker diamine oxidase (DOA) was significantly elevated in the high-sugar, high-fat, and high-protein groups compared with the control group (**Supplementary Fig. 2a**). Further observation of intestinal morphological changes showed that the intestinal structure was abnormal in high-sugar, high-fat, and high-protein groups, and inflammatory cell infiltration was observed in cecum and colon (**Supplementary Fig. 2b**). The above results show that high-sugar, high-fat, and high-protein diets can have a significant pro-inflammatory effect.


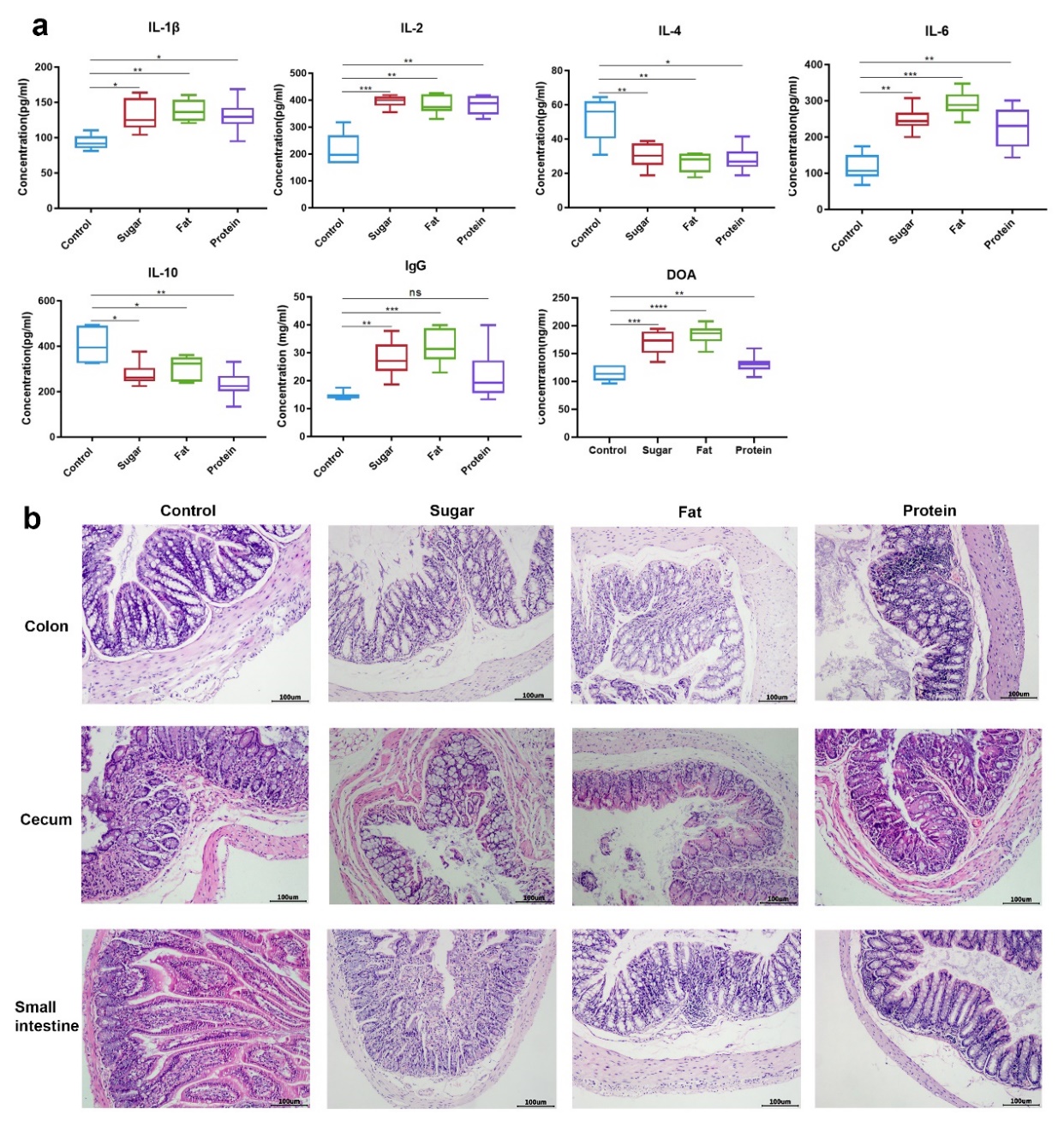


**Supplemental Fig. 2 Immune status of mice in each diet group.** Mouse serum immune cytokines, immunoglobulins, and intestinal permeability marker diamine oxidase (DOA) (**a**). H&E staining of pathological sections of each intestinal segment of mice (**b**).

**3 Analysis of receptor bacteria and intestinal resistant genome after intragastric administration of RP4 plasmid**

After intragastric administration of RP4 plasmid carrying exogenous ARGs, we screened the receptor bacteria containing exogenous ARGs (**Supplemental Fig.3a)** and sequenced them. The analysis of receptor bacteria at the phylum level showed that the receptor bacteria in each diet group were mainly distributed in *Proteobacteria*, *Firmicutes*, *Bacteroidetes*, *Deferibacters*, *Actinobacteria* and *Verrucomicrobia*. Among them, *Proteobacteria* accounted for 66.44% in high glucose group (S-P), 55.16% in high fat group (F-P), 68.72% in high protein group (P-P) and 51.97% in normal diet group (C-P) (**Supplemental Fig. 3b, c**). This is consistent with the results of ARB gavage, and the proportion of *Proteobacteria* is the highest. Further analysis of receptor bacteria at the genus level showed that in high-sugar group (S-P), exogenous ARGs were mainly enriched in *Ralstonia*, *Vibrio*, *Pseudoalteromonas*, *Bacteroides*, *Pseudomonas*, *Akkermansia*, *Mucispirillum*, *Colidextribacter*, *Lactobacillus*, *Streptococcus*, *Dubosiella*. In high-fat group (F-P), exogenous ARGs were mainly enriched in *Ralstonia*, *Mucispirillum*, *Colidextribacter*, *Odoribacter*, *Blautia*, *Vibrio*, *Lachnoclostridium*, *Akkermansia*, *Oscillibacter*. In high-protein group (P-P), exogenous ARGs were mainly enriched in *Ralstonia*, *Vibrio*, *Pseudoalteromonas*, *Pseudomonas*, *Streptococcus*, *Lactobacillus*, *Bacteroides*, *Mucispirillum*. In the normal diet group, exogenous ARGs were mainly enriched in *Ralstonia*, *Akkermansia*, *Parabacteroides*, *Mucispirillum*, *Rikenella*, *Dubosiella*, *Bacteroides*, *Colidextribacter*.

Compared with the dominant bacteria in the corresponding diet at the phylum level (top10) and the genus level (top35), some high abundance receptor bacteria in each diet group after administration of RP4 plasmid were the same as the dominant bacteria, indicating that some receptor bacteria came from the dominant bacteria. After adding plasmid, there are 7 kinds of receptor bacteria that are the same as the dominant bacteria at the phylum level, including *Proteobacteria*, *Bacteroides*, *Firmicutes*, *Actinomycetes*, *Verrucomicrobia*, *Acidobacterita* and *Deferribactes*. There are 15 kinds of receptor bacteria that are the same as the dominant bacteria at the genus level, including *Muscispirillum*, *Oscillibacter*, *Bacteroides*, *Akkermansia*, *Streptococcus*, *Lachnoclostridium*, *Rikenella*, *Helicobacter*, *Faecalibaculum*, *Bifidobacterium*, *Ralstonia*, *Odoribacter*, *Alistipes*, *Roseburia*, *Parabacteroides* (**Supplemental Fig. 3d, e**).

By analyzing the resistance group after intragastric administration of RP4 plasmid, it was found that the resistance genes were mainly distributed in *Firmicutes*, *Proteobacteria*, *Bacteroides*, *Chlamydia*, *Actinomycetes* and *Verrucomicrobia* (**Supplemental Fig.3f**). Among them, *Firmicutes* are the most distributed, followed by *Proteobacteria* and *Bacteroidetes*. Consistent with the results of ARB by gavage, the change trend of *Firmicutes* and *Bacteroidetes* in each group was inconsistent before and after the addition of RP4 plasmid, while the Proteus in each diet group increased significantly after the addition of RP4 plasmid, and the number of *Proteobacteria* in the high-protein group was the most (**Supplemental Fig. 3g-i**).


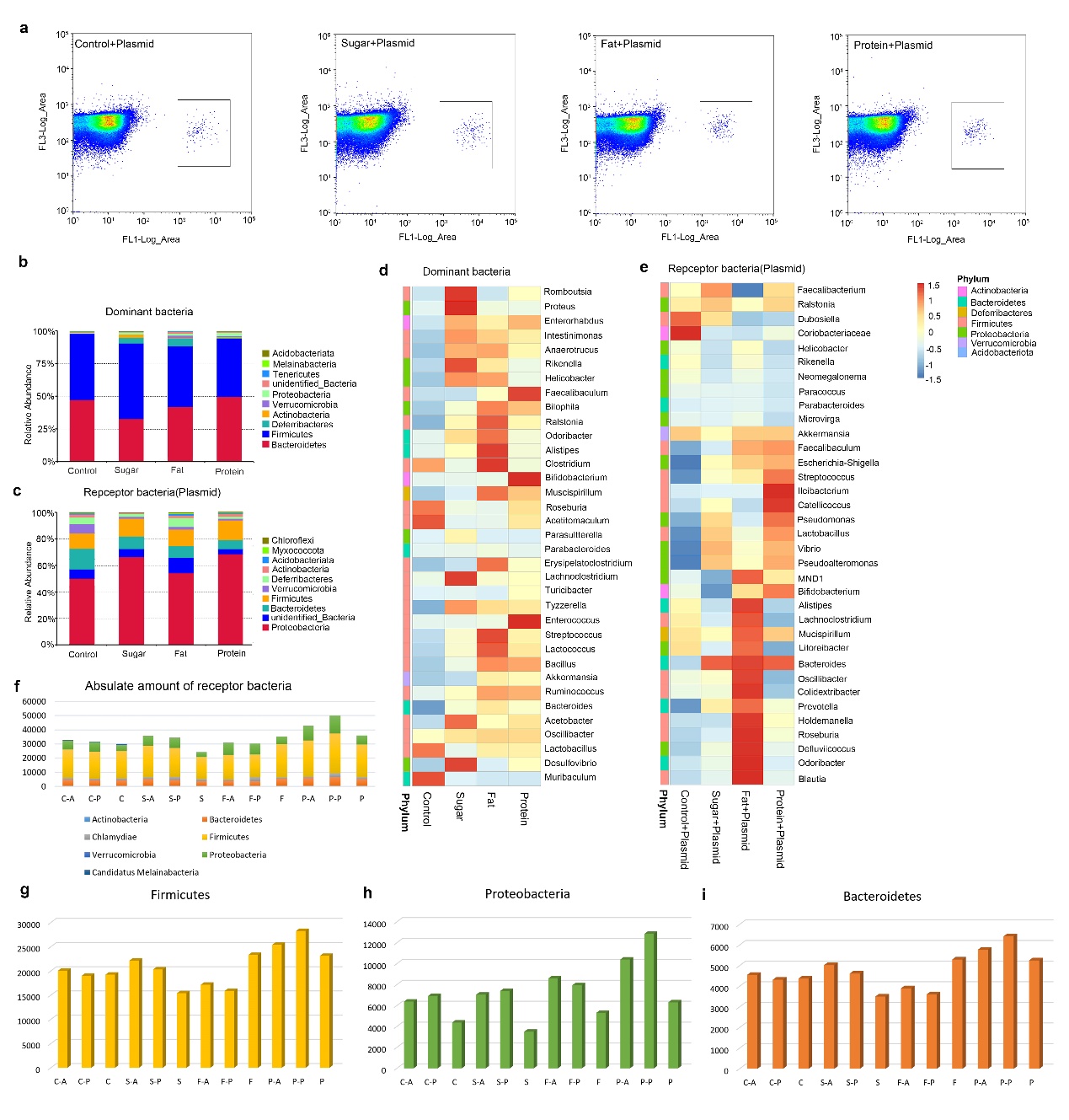


**Supplemental Fig.3 Distribution of receptor bacteria and intestinal resistance genome of RP4 plasmid by gavage.** Flow cytometry was used to select receptor bacteria with green fluorescence, and positive bacteria were collected in a small square frame (**a**). Top 10 species distribution histogram at the phylum level for receptor bacteria and dominant bacteria in each diet group (**b, c**). Top 35 species distribution heatmap at the genus level for receptor bacteria and dominant bacteria in each diet group, different colors on the left of the heat map indicate the phylum to which each genus belongs (**d, e**). Absolute number of different bacteria containing ARGs in each group (**f-i**)

**4 16S rRNA Sequencing**

4.1 Extraction of genome DNA

Total genome DNA from samples was extracted using CTAB method. DNA concentration and purity was monitored on 1% agarose gels. According to the concentration, DNA was diluted to 1ng/µL using sterile water.

4.2 Amplicon Generation

16S rRNA genes of 16S V4 regions were amplified used specific primer 515F-806R. All PCR reactions were carried out with 15 µL of Phusion® High-Fidelity PCR Master Mix (New England Biolabs); 0.2 µM of forward and reverse primers, and about 10 ng template DNA. Thermal cycling consisted of initial denaturation at 98℃ for 1 min, followed by 30 cycles of denaturation at 98℃ for 10 s, annealing at 50℃ for 30 s, and elongation at 72℃ for 30 s. Finally 72℃ for 5 min.

4.3 PCR Products quantification and qualification

Mix same volume of 1X loading buffer (contained SYB green) with PCR products and

operate electrophoresis on 2% agarose gel for detection. PCR products was mixed in equidensity ratios. Then, mixture PCR products was purified with Qiagen Gel Extraction Kit (Qiagen, Germany).

4.4 Library preparation and sequencing

Sequencing libraries were generated using TruSeq DNA PCR-Free Sample Preparation Kit (Illumina, USA) following manufacturer's recommendations and index codes were added. The library quality was assessed on the Qubit@ 2.0 Fluorometer (Thermo Scientific) and Agilent Bioanalyzer 2100 system. At last, the library was sequenced on an Illumina NovaSeq platform and 250 bp paired-end reads were generated.

4.5 Paired-end reads assembly and quality control

4.5.1 Data split: Paired-end reads was assigned to samples based on their unique barcode and truncated by cutting off the barcode and primer sequence.

4.5.2 Sequence assembly: Paired-end reads were merged using FLASH (V1.2.7,http://ccb.jhu.edu/software/FLASH/) ^5^, a very fast and accurate analysis tool, which was designed to merge paired-end reads when at least some of the reads overlap the read generated from the opposite end of the same DNA fragment, and the splicing sequences were called raw tags.

4.5.3 Data Filtration: Quality filtering on the raw tags were performed under specific filtering conditions to obtain the high-quality clean tags^6^ according to the QIIME (v2.0, <http://qiime2.org/>) ^7^, quality controlled process.

4.5.4 Chimera removal: The tags were compared with the reference database (Silva database, https://www.arb-silva.de/) using UCHIME algorithm (UCHIME Algorithm, <http://www.drive5.com/usearch/manual/uchime_algo.html>)^8^ to detect chimera sequences, and then the chimera sequences were removed^9^. Then the Effective Tags finally obtained.

4.6 OTU cluster and Species annotation

4.6.1 OTU Production: Sequences analysis were performed by Uparse software (Uparse v7.0.1001，<http://drive5.com/uparse/>) ^10^. Sequences with ≥97% similarity were assigned to the same OTUs.Representative sequence for each OTU was screened for further annotation.

4.6.2 Species annotation: For each representative sequence, the Silva Database (http://www.arb-silva.de/)^11^ was used based on Mothur algorithm to annotate taxonomic information.

4.6.3 Phylogenetic relationship Construction: In order to study phylogenetic relationship of different OTUs, and the difference of the dominant species in different samples(groups), multiple sequence alignment were conducted using the MUSCLE software (Version 3.8.31, <http://www.drive5.com/muscle/>)^12^.

4.6.4 Data Normalization

OTUs abundance information were normalized using a standard of sequence number corresponding to the sample with the least sequences. Subsequent analysis of alpha diversity and beta diversity were all performed basing on this output normalized data.

4.7 Alpha Diversity

Alpha diversity is applied in analyzing complexity of species diversity for a sample through 6 indices, including Observed-species, Chao1, Shannon, Simpson, ACE, Good-coverage. All this indices in our samples were calculated with QIIME (Version 2.0) and displayed with R software(Version 2.15.3).

Two indices were selected to identify Community richness:

Chao - the Chao1 estimator (http://www.mothur.org/wiki/Chao).

ACE - the ACE estimator (<http://www.mothur.org/wiki/Ace>).

Two indices were used to identify Community diversity:

Shannon - the Shannon index (http://www.mothur.org/wiki/Shannon).

Simpson - the Simpson index (http://www.mothur.org/wiki/Simpson).

One indice to characterized Sequencing depth:

Coverage - the Good’s coverage (http://www.mothur.org/wiki/Coverage).
4.8 Beta Diversity

Beta diversity analysis was used to evaluate differences of samples in species complexity, Beta diversity on both weighted and unweighted unifrac were calculated by QIIME software (Version 2.0).

Cluster analysis was preceded by principal component analysis (PCA), which was applied to reduce the dimension of the original variables using the FactoMineR package and ggplot2 package in R software (Version 2.15.3).

Principal Coordinate Analysis (PCoA) was performed to get principal coordinates and

visualize from complex, multidimensional data. A distance matrix of weighted or unweighted unifrac among samples obtained before was transformed to a new set of orthogonal axes, by which the maximum variation factor is demonstrated by first principal coordinate, and the second maximum one by the second principal coordinate, and so on. PCoA analysis was displayed by WGCNA package, stat packages and ggplot2 package in R software (Version 2.15.3).

**5 Metabolomics**

5.1 Metabolites Extraction

Tissues (100 mg) were individually grounded with liquid nitrogen and the homogenate was resuspended with prechilled 80% methanol and 0.1% formic acid by well vortex. The samples were incubated on ice for 5 min and then were centrifuged at 15,000 rpm, 4°C for 5 min. Some of supernatant was diluted to final concentration containing 53% methanol by LC-MS grade water. The samples were subsequently transferred to a fresh Eppendorf tube and then were centrifuged at 15000 g, 4°C for 10 min. Finally, the supernatant was injected into the LC-MS/MS system analysis^13^. PS: Liquid sample (100 μL) and prechilled methanol (400 μL) were mixed by well vortexing^14^. Cell sample and 4 times prechilled 80% methanol were mixed by well vortexing, and then sonicated for 6 min. Repeating this step once again and then operating the same steps as above^15, 16^.

5.2 UHPLC-MS/MS Analysis

UHPLC-MS/MS analyses were performed using a Vanquish UHPLC system (Thermo Fisher, Germany) coupled with an Orbitrap Q ExactiveTM HF mass spectrometer (Thermo Fisher, Germany) in Novogene Co., Ltd. (Beijing, China). Samples were injected onto a Hypesil Gold column (100×2.1 mm, 1.9μm) using a 17-min linear gradient at a flow rate of 0.2 mL/min. The eluents for the positive polarity mode were eluent A (0.1% FA in Water) and eluent B (Methanol).The eluents for the negative polarity mode were eluent A (5 mM ammonium acetate, pH 9.0) and eluent B (Methanol).The solvent gradient was set as follows: 2% B, 1.5 min; 2-100% B, 12.0 min; 100% B, 14.0 min；100-2% B, 14.1 min；2% B, 17 min. Q ExactiveTM HF mass spectrometer was operated in positive/negative polarity mode with spray voltage of 3.2 kV, capillary temperature of 320°C, sheath gas flow rate of 40 arb and aux gas flow rate of 10 arb.

5.3 Data processing and metabolite identification

The raw data files generated by UHPLC-MS/MS were processed using the Compound Discoverer 3.1 (CD3.1, Thermo Fisher) to perform peak alignment, peak picking, and quantitation for each metabolite. The main parameters were set as follows: retention time tolerance, 0.2 minutes; actual mass tolerance, 5ppm; signal intensity tolerance, 30%; signal/noise ratio, 3; and minimum intensity, 100，000. After that, peak intensities were normalized to the total spectral intensity. The normalized data was used to predict the molecular formula based on additive ions, molecular ion peaks and fragment ions. And then peaks were matched with the mzCloud (<https://www.mzcloud.org/)，mzVault> and MassList database to obtain the accurate qualitative and relative quantitative results. Statistical analyses were performed using the statistical software R (R version R-3.4.3), Python (Python 2.7.6 version) and CentOS (CentOS release 6.6), When data were not normally distributed, normal transformations were attempted using of area normalization method.

5.4 Data Analysis

These metabolites were annotated using the KEGG database (<https://www.genome.jp/kegg/pathway.html>), HMDB database (https://hmdb.ca/ metabolites) and LIPID Maps database (http://www.lipidmaps.org/). Principal components analysis (PCA) and Partial least squares discriminant analysis (PLS-DA) were performed at metaX (a flexible and comprehensive software for processing metabolomics data). We applied univariate analysis (t-test) to calculate the statistical significance (P-value). The metabolites with VIP > 1 and p-value< 0.05 and fold change≥ 2 or FC≤ 0.5 were considered to be differential metabolites. Volcano plots were used to filter metabolites of interest which based on log2 (FoldChange) and -log10 (p-value) of metabolites. For clustering heat maps, the data were normalized using z-scores of the intensity areas of differential metabolites and were ploted by Pheatmap package in R language. The correlation between differential metabolites were analyzed by R (method is Pearson). Statistically significant of correlation between differential metabolites were calculated by cor.mtest (in R language. P-value < 0.05) was considered as statistically significant and correlation plots were ploted by corrplot package in R language. The functions of these metabolites and metabolic pathways were studied using the KEGG database. The metabolic pathways enrichment of differential metabolites was performed, when ratio were satisfied by x/n > y/N, metabolic pathway were considered as enrichment, when P-value of metabolic pathway < 0.05, metabolic pathway were considered as statistically significant enrichment.

**6 Metagenomic**

Metagenomic analysis can more truly reflect the microbial composition and interaction in the sample, and study the metabolic pathway and gene function at the molecular level^17^.

6.1. Sequencing results pretreatment

6.1.1 Preprocessing the Raw Data obtained from the Illumina HiSeq sequencing platform using Readfq (V8, https://github.com/cjfields/readfq) was conducted to acquire the Clean Data for subsequent analysis. The specific processing steps are as follows: a) remove the reads which contain low quality bases (default quality threshold value ≤ 38) above a certain portion (default length of 40 bp); b) remove the reads in which the N base has reached a certain percentage (default length of 10 bp); c) remove reads which shared the overlap above a certain portion with Adapter (default length of 15 bp).

6.1.2 Considering the possibility of host pollution may exist in samples, Clean Data need to be blast to the host database which default using Bowtie2.2.4 software (Bowtie2.2.4, http://bowtiebio.sourceforge.net/bowtie2/index.shtml) to filter the reads that are of host origin, the parameters^18^ are as follows : --end-to-end, --sensitive, -I 200, -X 400.

6.2 Metagenome Assembly

6.2.1 Single sample assembly:

To the samples taken from non-complex environment, such as intestine, faeces and so on, the Clean Data is assembled and analyst^19^ by SOAPdenovo software (V2.04, <http://soap.genomics.org.cn/soapdenovo.html>), the parameters^20^ are as follows: -d 1, -M 3, -R, -u, -F, -K 55; To the samples taken from complex environment, such as water, soil and so on, MEGAHIT software (v1.0.4-beta) could be used to assemble the Clean Data and the parameters^21^ are -presets meta-large (-- min-count 2 --k-min 27 --k-max 87 --k-step 10; then interrupted the assembled Scaftigs from N connection and leave the Scaftigs without N^22^. All samples’ Clean Data are compared to each Scaffolds respectively by Bowtie2.2.4 software to acquire the PE reads not used and the parameters are: --end-to-end, --sensitive, -I 200, -X 400.

6.2.2 Mixed assembly:

all the reads not used in the forward step of all samples are combined and then use the software of SOAPdenovo (V2.04 ) / MEGAHIT (v1.0.4-beta) for mixed assembly with the parameters same as single assembly; Break the mixed assembled Scaffolds from N connection and obtained the Scaftigs. Filter the fragment shorter than 500 bp in all of Scaftigs for statistical analysis both generated from single or mixed assembly.

6.3 Gene prediction and abundance analysis

6.3.1 The Scaftigs (≥ 500 bp) assembled from both single and mixed are all predicted the ORF by MetaGeneMark (V2.10, http://topaz.gatech.edu/GeneMark/) software, and filtered the length information shorter than 100 nt^23^ from the predicted result with default parameters.

6.3.2 For ORF predicted, CD-HIT^24^ oftware (V4.5.8, http://www.bioinformatics.org/cd-hit ) is adopted to redundancy and obtain the unique initial gene catalogue (the genes here refers to the nucleotide sequences coded by unique and continuous genes^25^), the parameters option are -c 0.95, -G 0, -a S 0.9, -g 1, -d 0.

6.3.3 The Clean Data of each sample is mapped to initial gene catalogue using Bowtie2.2.4 and get the number of reads to which genes mapped in each sample with the parameter setting^26^ are -- end-to-end, --sensitive, -I 200, -X 400. Filter the gene which the number of reads ≤ 2^27^ in each sample and obtain the gene catalogue (Unigenes) eventually used for subsequently analysis.

6.3.4 Based on the number of mapped reads and the length of gene, statistic the abundance information of each gene in each sample. The format is as follow, r represents the number of reads mapped to the genes and L represents gene’s length^28^.

6.3.5 The basic information statistic, core-pan gene analysis, correlation analysis of samples and venn figure analysis of number of genes are all based on the abundance of each gene in each sample in gene catalogue.

6.4 Taxonomy prediction

6.4.1 DIAMOND^29^ software (V0.9.9, https://github.com/bbuchfink/diamond/) is used to blast the Unigenes to the sequences of Bacteria, Fungi, Archaea and Viruses which are all extracted from the NR database (Version: 2018-01-02, https://www.ncbi.nlm.nih.gov/) of NCBI with the parameter setting are blast p-e 1e-5.

6.4.2 For the finally aligned results of each sequence, as each sequence may have multiple aligned results, choose the result of which the e value ≤ the smallest e value * 10 to take the LCA algorithm which is applied to system classification of MEGAN^30^ software to make sure the species annotation information of sequences.

6.4.3 The table containing the number of genes and the abundance information of each sample in each taxonomy hierarchy (kingdom, phylum, class, order, family, genus, species) are obtained based on the LCA annotation result and the gene abundance table. The abundance of a specie in one sample equal the sum of the gene abundance annotated for the specie; the gene number of a specie in a sample equal the number of genes whose abundance are nonzero.

6.4.4 Krona analysis, the exhibition of generation situation of relative abundance, the exhibition of abundance cluster heat map, PCA^31^ (R ade4 package, Version 2.15.3) and NMDS^32^ (R vegan package, Version 2.15.3) decrease-dimension analysis are based on the abundance table of each taxonomic hierarchy. The difference between groups is tested by Anosim analysis (R vegan package, Version 2.15.3). Metastats and LEfSe analysis are used to look for the different species between groups. Permutation test between groups is used in Metastats analysis for each taxonomy and get the P value, then use Benjamini and Hochberg False Discovery Rate to correct P value and acquire q value^33^. LEfSe analysis is conducted by LEfSe software (the default LDA score is 3)^34^; Finally, random forest (RandoForest) (R pROC and randomForest packages, Version 2.15.3) was used to construct a random forest model. Screen out important species by MeanDecreaseAccuracy and MeanDecreaseGin, then cross-validate each model (default 10 times) and plot the ROC curve.

6.5 Common functional database annotations

6.5.1 Adopt DIAMOND software (V0.9.9) to blast Unigenes to functional database with the parameter setting of blastp, -e 1e-5. Functional database exclude KEGG^35^ database (Version 2018-01-01, http://www.kegg.jp/kegg/), eggNOG^36^ database (Version 4.5, http://eggnogdb.embl.de/#/app/home), CAZy^37^ database (Version 201801, http://www.cazy.org/). For each sequence’s blast result, the best Blast Hit is used for subsequent analysis^38^.

6.5.2 Statistic of the relative abundance of different functional hierarchy, the relative abundance of each functional hierarchy equal the sum of relative abundance annotated to that functional level.

6.5.3 Based on the function annotation result and gene abundance table, the gene number table of each sample in each taxonomy hierarchy is obtained. The gene number of a function in a sample equal the gene number that annotated to this function and the abundance is nonzero.

6.5.4 Based on the abundance table of each taxonomy hierarchy, not only the counting of annotated gene numbers, the exhibition of the general relative abundance situation, the exhibition of abundance cluster heat map and the decrease-dimension analysis of PCA and NMDS are conducted, but also the Anosim analysis of the difference between groups (inside) based on functional abundance, comparative analysis of metabolic pathways, the Metatat and LEfSe analysis of functional difference between groups are performed.

6.6 Resistance gene annotation

6.6.1 Use Resistance Gene Identifier (RGI) software to align the Unigenes to CARD database (https://card.mcmaster.ca/)^39^ with the parameter settting are blastp, evalue ≤ 1e-30.

6.6.2 Based on the aligned result, count the relative abundance of ARO.

6.6.3 Based on the abundance of ARO, the abundance bar charts, the abundance cluster heatmap and the resistance genes’ number difference between groups are displayed. In the same way. The resistance genes’ abundance distribution in each samples, the species attribution analysis of resistance genes and the resistance mechanism of resistance genes analysis are also conducted.

**Sequence information of double fluorescent labeled donor bacteria**

1. Gene sequence to be inserted:

TTGACAATTAATCATCGGCTCGTATAATGCCTCTAGAAATAATTTTGTTTAACTTTAAGAAGGAGATATACCATGGAAGCTTATGGTTTCCAAGGGCGAGGAGGATAACATGGCTATCATTAAAGAGTTCATGCGCTTCAAAGTTCACATGGAGGGTTCTGTTAACGGTCACGAGTTCGAGATCGAAGGCGAAGGCGAGGGCCGTCCGTATGAAGGCACCCAGACCGCCAAACTGAAAGTGACTAAAGGCGGCCCGCTGCCTTTTGCGTGGGACATCCTGAGCCCGCAATTTATGTACGGTTCTAAAGCGTATGTTAAACACCCAGCGGATATCCCGGACTATCTGAAGCTGTCTTTTCCGGAAGGTTTCAAGTGGGAACGCGTAATGAATTTTGAAGATGGTGGTGTCGTGACCGTCACTCAGGACTCCTCCCTGCAGGATGGCGAGTTCATCTATAAAGTTAAACTGCGTGGTACTAATTTTCCATCTGATGGCCCGGTGATGCAGAAAAAGACGATGGGTTGGGAGGCGTCTAGCGAACGCATGTATCCGGAAGATGGTGCGCTGAAAGGCGAAATTAAACAGCGCCTGAAACTGAAAGATGGCGGCCATTATGACGCTGAAGTGAAAACCACGTACAAAGCCAAGAAACCTGTGCAGCTGCCTGGCGCGTACAATGTGAATATTAAACTGGACATCACCTCTCATAATGAAGATTATACGATCGTAGAGCAATATGAGCGCGCGGAGGGTCGTCATTCTACCGGTGGCATGGATGAGCTGTACAAATAA

2. To replace the genome sequence of msbB:

ATGGAAACGAAAAAAAATAATAGCGAATACATTCCTGAGTTTGATAAATCCTTTCGCCACCCGCGCTACTGGGGAGCATGGCTGGGCGTAGCAGCGATGGCGGGTATCGCTTTAACGCCGCCAAAGTTCCGTGATCCCATTCTGGCACGGCTGGGACGTTTTGCCGGACGACTGGGAAAAAGCTCACGCCGTCGTGCGTTAATCAATCTGTCGCTCTGCTTTCCAGAACGTAGTGAAGCTGAACGCGAAGCGATTGTAGATGAGATGTTTGCCACCGCGCCGCAAGCGATGGTAATGATGGCTGAGTTGGCAATACGCGGGCCGGAGAAAATTCAGCCGCGCGTTGACTGGCAAGGGCTGGAGATCATCGAAGAGATGCGGCGTAATAACGAGAAAGTGATTTTTCTGGTGCCGCACGGTTGGGCCGTCGATATTCCTGCCATGCTGATGGCCTCGCAAGGGCAGAAAATGGCAGCGATGTTCCATAATCAGGGCAACCCGGTTTTTGATTATGTCTGGAACACGGTGCGCCGTCGCTTTGGTGGTCGTCTGCATGCGAGAAATGATGGTATTAAACCATTCATCCAGTCGGTACGTCAGGGTTACTGGGGATATTATTTACCCGATCAGGATCATGGCCCAGAACACAGCGAATTTGTTGATTTCTTTGCCACCTATAAAGCGACGTTGCCCGCGATTGGTCGTTTGATGAAAGTGTGCCGTGCGCGCGTTGTACCGCTGTTTCCGATTTATGATGGCAAGACGCATCGTCTGACGATTCAGGTGCGCCCACCGATGGATGATCTGTTAGAGGCGGATGATCATACGATTGCGCGGCGGATGAATGAAGAAGTCGAGATTTTTGTTGGTCCGCGACCAGAACAATACACCTGGATATTAAAATTGCTGAAAACTCGCAAACCGGGCGAAATCCAACCGTATAAGCGCAAAGATCTTTATCCCATCAAATAA

3. The sequence of msbB was obtained by NCBI:

GGCGAAACAGTTCCGTATCTCATCTAACTTTAACCCGCGTCGTACTAATCCGGTGACCGGTCGCGTTGCGCCACACAGAGGTGTTGATTTTGCCATGCCACAAGGTACGCCAGTGCTTTCAGTGGGTGACGGTGAAGTGGTGGTTGCCAAACGCAGTGGCGCAGCAGGTTATTATGTGGCTATTCGTCATGGTCGCAGCTACACCACGCGTTATATGCACTTGCGCAAGATCCTCGTGAAACCGGGACAGAAGGTGAAACGTGGCGACCGTATCGCGCTTTCCGGTAATACCGGACGTTCAACCGGGCCGCATCTGCACTATGAAGTATGGATAAACCAGCAGGCCGTAAACCCGCTGACGGCAAAACTGCCGCGTACCGAAGGGCTGACCGGCTCCGATCGTCGCGAATTCCTGGCACAGGCCAAAGAGATTGTGCCGCAGCTACGGTTTGATTAATTAACATCCATTCGCAGCCGGTACGCAGTCAGTACCGGCTTTTATTTGGTGCGGGGCAAGTTGCGCCGCTACACTATCACCAGATTGATTTTTGCCTTATCCGAAACTGGAAAAGCATGGAAACGAAAAAAAATAATAGCGAATACATTCCTGAGTTTGATAAATCCTTTCGCCACCCGCGCTACTGGGGAGCATGGCTGGGCGTAGCAGCGATGGCGGGTATCGCTTTAACGCCGCCAAAGTTCCGTGATCCCATTCTGGCACGGCTGGGACGTTTTGCCGGACGACTGGGAAAAAGCTCACGCCGTCGTGCGTTAATCAATCTGTCGCTCTGCTTTCCAGAACGTAGTGAAGCTGAACGCGAAGCGATTGTAGATGAGATGTTTGCCACCGCGCCGCAAGCGATGGTAATGATGGCTGAGTTGGCAATACGCGGGCCGGAGAAAATTCAGCCGCGCGTTGACTGGCAAGGGCTGGAGATCATCGAAGAGATGCGGCGTAATAACGAGAAAGTGATTTTTCTGGTGCCGCACGGTTGGGCCGTCGATATTCCTGCCATGCTGATGGCCTCGCAAGGGCAGAAAATGGCAGCGATGTTCCATAATCAGGGCAACCCGGTTTTTGATTATGTCTGGAACACGGTGCGCCGTCGCTTTGGTGGTCGTCTGCATGCGAGAAATGATGGTATTAAACCATTCATCCAGTCGGTACGTCAGGGTTACTGGGGATATTATTTACCCGATCAGGATCATGGCCCAGAACACAGCGAATTTGTTGATTTCTTTGCCACCTATAAAGCGACGTTGCCCGCGATTGGTCGTTTGATGAAAGTGTGCCGTGCGCGCGTTGTACCGCTGTTTCCGATTTATGATGGCAAGACGCATCGTCTGACGATTCAGGTGCGCCCACCGATGGATGATCTGTTAGAGGCGGATGATCATACGATTGCGCGGCGGATGAATGAAGAAGTCGAGATTTTTGTTGGTCCGCGACCAGAACAATACACCTGGATATTAAAATTGCTGAAAACTCGCAAACCGGGCGAAATCCAACCGTATAAGCGCAAAGATCTTTATCCCATCAAATAAAAAATGCCTCTCGCGAGGAGAGGCCTTCGACTGATGATAAGTTCAAGTTTGCTTCAGAAGATTCGAAATCTGTTGAATTATCATTGAACTGTAGGCCGGATGAGGCGTTTTCGCCGCATCCGGCAACGTACTTACTCTACCGTTAAAATACGCGTGGTATTAGTAGAACCCACGGTACTCATCACGTCGCCCTGGGTGACAATCACCAGGTCACCAGACATCAAGTAACCTTTATCGCGCAGCAGATTAACCGCTTCGCTGGCAGCCGCTACGCCGTCATTAGCGCTATCAAAGTGCACCGGCGTAACGCCGCGATAGAGAGCAGTCAGGTTCAGCGTACGTTCATGGCGTGACATGGCGAAAATTGGCAGACCAGAGCTGATACGGGAGGTCATCAGCGCGGTACGACCCGATTCGGTCATGGTGATGATCGCCGTAACGCCTTTCAGGTGGTTAGCCGCGTACATTGCTGACATGGCAATAGCTTCTTCCACATTGTCGAACTGAACGTCCAGACGGTGTTTAGAAACGTTGATGCTCG

4. Sequence analysis of the upstream homologous arm of E5 msbB:

GCGAAACAGTTCCGTATCTCGTCTAACTTTAACCCGCGTCGTACTAATCCGGTGACCGGTCGCGTTGCACCACACAGAGGTGTTGATTTCGCCATGCCACAAGGTACGCCAGTGCTTTCAGTGGGTGACGGTGAAGTGGTGGTTGCCAAACGCAGTGGTGCAGCAGGTTATTATGTGGCTATTCGTCATGGTCGCAGCTACACCACGCGTTATATGCACTTGCGCAAGATTCTGGTGAAACCGGGACAGAAGGTGAAACGTGGCGACCGTATCGCGCTTTCCGGTAATACCGGACGTTCAACCGGGCCGCATCTGCACTATGAAGTATGGATAAACCAGCAGGCCGTAAACCCGCTGACGGCAAAACTGCCGCGTACCGAAGGGCTGACCGGCTCCGATCGTCGCGAATTCCTGGCGCAGGCCAAAGAGATTGTGCCGCAGCTACGGTTTGATTAATTAACATCCATTCGCAGCCGGTACGAAGTCAGTACCGGCTTTTTTTATTTGGTGCGGGGCAAGTTGCGCCGCTACACTATCACCAGATTGATTTTTGCCTTATCCGAAACTGGAAAAGC

5. Sequence analysis of the downstream homologous arm of E5 msbB:

CAAATAAAAAATGCCTCTCGCGAGGAGAGGCCTTCGACTGATGATAAGTTCAAGTTTGCTTCAGAAGATTCGAAATCTGTTGAATTATCATTGAACTGTAGGCCGGATGAGGCGTTTTCGCCGCATCCGGCAACGTACTTACTCTACCGTTAAAATACGCGTGGTATTAGTAGAACCCACGGTACTCATCACGTCGCCCTGGGTGACAATCACCAGGTCACCAGACATCAAGTAACCTTTATCGCGCAGCAGATTAACCGCTTCGCTGGCAGCTGCTACACCGTCATTAGCGCTATCAAAGTGCACCGGCGTAACGCCGCGATAGAGAGCTGTCAGGTTCAGCGTACGTTCATGGCGTGACATGGCGAAAATTGGCAGACCAGAGCTGATACGGGAGGTCATCAGCGCGGTACGACCCGATTCGGTCATGGTGATGATCGCCGTAACGCCTTTCAGGTGGTTAGCCGCGTACATTGCTGACATGGCAATGGCTTCTTCCACATTGTCGAACTGAACATCCAGACGGTGTTTAGAAACGTTGATGCTCG

**Reference**

1. Li MC, Tang HJ, Wu CJ, Wang SW, Su SL, Liu WL, et al. Species identification and antifungal susceptibility of uncommon blood yeast isolates. J Microbiol Immunol Infect 2021.

2. Cherkaoui A, Fischer A, Azam N, Riat A, Schrenzel J. A comparison of Sensititre Anaerobe MIC plate with ATB ANA(R) test for the routine susceptibility testing of common anaerobe pathogens. Eur J Clin Microbiol Infect Dis 2018; 37:2279-84.

3. Dingle TC, Butler-Wu SM. Maldi-tof mass spectrometry for microorganism identification. Clin Lab Med 2013; 33:589-609.

4. Wu Y, Liu C, Li WG, Xu JL, Zhang WZ, Dai YF, et al. Independent Microevolution Mediated by Mobile Genetic Elements of Individual Clostridium difficile Isolates from Clade 4 Revealed by Whole-Genome Sequencing. mSystems 2019; 4.

5. Magoc T, Salzberg SL. FLASH: fast length adjustment of short reads to improve genome assemblies. Bioinformatics 2011; 27:2957-63.

6. Bokulich NA, Subramanian S, Faith JJ, Gevers D, Gordon JI, Knight R, et al. Quality-filtering vastly improves diversity estimates from Illumina amplicon sequencing. Nat Methods 2013; 10:57-9.

7. Bokulich NA, Kaehler BD, Rideout JR, Dillon M, Bolyen E, Knight R, et al. Optimizing taxonomic classification of marker-gene amplicon sequences with QIIME 2's q2-feature-classifier plugin. Microbiome 2018; 6:90.

8. Edgar RC, Haas BJ, Clemente JC, Quince C, Knight R. UCHIME improves sensitivity and speed of chimera detection. Bioinformatics 2011; 27:2194-200.

9. Haas BJ, Gevers D, Earl AM, Feldgarden M, Ward DV, Giannoukos G, et al. Chimeric 16S rRNA sequence formation and detection in Sanger and 454-pyrosequenced PCR amplicons. Genome Res 2011; 21:494-504.

10. Edgar RC. UPARSE: highly accurate OTU sequences from microbial amplicon reads. Nat Methods 2013; 10:996-8.

11. Quast C, Pruesse E, Yilmaz P, Gerken J, Schweer T, Yarza P, et al. The SILVA ribosomal RNA gene database project: improved data processing and web-based tools. Nucleic Acids Res 2013; 41:D590-6.

12. Edgar RC. MUSCLE: multiple sequence alignment with high accuracy and high throughput. Nucleic Acids Res 2004; 32:1792-7.

13. Want EJ, O'Maille G, Smith CA, Brandon TR, Uritboonthai W, Qin C, et al. Solvent-dependent metabolite distribution, clustering, and protein extraction for serum profiling with mass spectrometry. Anal Chem 2006; 78:743-52.

14. Barri T, Dragsted LO. UPLC-ESI-QTOF/MS and multivariate data analysis for blood plasma and serum metabolomics: effect of experimental artefacts and anticoagulant. Anal Chim Acta 2013; 768:118-28.

15. Sellick CA, Hansen R, Stephens GM, Goodacre R, Dickson AJ. Metabolite extraction from suspension-cultured mammalian cells for global metabolite profiling. Nat Protoc 2011; 6:1241-9.

16. Yuan M, Breitkopf SB, Yang X, Asara JM. A positive/negative ion-switching, targeted mass spectrometry-based metabolomics platform for bodily fluids, cells, and fresh and fixed tissue. Nat Protoc 2012; 7:872-81.

17. Tringe SG, von Mering C, Kobayashi A, Salamov AA, Chen K, Chang HW, et al. Comparative metagenomics of microbial communities. Science 2005; 308:554-7.

18. Law J, Jovel J, Patterson J, Ford G, O'Keefe S, Wang W, et al. Identification of hepatotropic viruses from plasma using deep sequencing: a next generation diagnostic tool. PLoS One 2013; 8:e60595.

19. Luo R, Liu B, Xie Y, Li Z, Huang W, Yuan J, et al. SOAPdenovo2: an empirically improved memory-efficient short-read de novo assembler. Gigascience 2012; 1:18.

20. Brum JR, Ignacio-Espinoza JC, Roux S, Doulcier G, Acinas SG, Alberti A, et al. Ocean plankton. Patterns and ecological drivers of ocean viral communities. Science 2015; 348:1261498.

21. Li D, Liu CM, Luo R, Sadakane K, Lam TW. MEGAHIT: an ultra-fast single-node solution for large and complex metagenomics assembly via succinct de Bruijn graph. Bioinformatics 2015; 31:1674-6.

22. Nielsen HB, Almeida M, Juncker AS, Rasmussen S, Li J, Sunagawa S, et al. Identification and assembly of genomes and genetic elements in complex metagenomic samples without using reference genomes. Nat Biotechnol 2014; 32:822-8.

23. Zeller G, Tap J, Voigt AY, Sunagawa S, Kultima JR, Costea PI, et al. Potential of fecal microbiota for early-stage detection of colorectal cancer. Mol Syst Biol 2014; 10:766.

24. Fu L, Niu B, Zhu Z, Wu S, Li W. CD-HIT: accelerated for clustering the next-generation sequencing data. Bioinformatics 2012; 28:3150-2.

25. Bahram M, Hildebrand F, Forslund SK, Anderson JL, Soudzilovskaia NA, Bodegom PM, et al. Structure and function of the global topsoil microbiome. Nature 2018; 560:233-7.

26. Li J, Jia H, Cai X, Zhong H, Feng Q, Sunagawa S, et al. An integrated catalog of reference genes in the human gut microbiome. Nat Biotechnol 2014; 32:834-41.

27. Qin J, Li Y, Cai Z, Li S, Zhu J, Zhang F, et al. A metagenome-wide association study of gut microbiota in type 2 diabetes. Nature 2012; 490:55-60.

28. Le Chatelier E, Nielsen T, Qin J, Prifti E, Hildebrand F, Falony G, et al. Richness of human gut microbiome correlates with metabolic markers. Nature 2013; 500:541-6.

29. Buchfink B, Xie C, Huson DH. Fast and sensitive protein alignment using DIAMOND. Nat Methods 2015; 12:59-60.

30. Huson DH, Mitra S, Ruscheweyh HJ, Weber N, Schuster SC. Integrative analysis of environmental sequences using MEGAN4. Genome Res 2011; 21:1552-60.

31. Avershina E, Frisli T, Rudi K. De novo semi-alignment of 16S rRNA gene sequences for deep phylogenetic characterization of next generation sequencing data. Microbes Environ 2013; 28:211-6.

32. Noval Rivas M, Burton OT, Wise P, Zhang YQ, Hobson SA, Garcia Lloret M, et al. A microbiota signature associated with experimental food allergy promotes allergic sensitization and anaphylaxis. J Allergy Clin Immunol 2013; 131:201-12.

33. White JR, Nagarajan N, Pop M. Statistical methods for detecting differentially abundant features in clinical metagenomic samples. PLoS Comput Biol 2009; 5:e1000352.

34. Segata N, Izard J, Waldron L, Gevers D, Miropolsky L, Garrett WS, et al. Metagenomic biomarker discovery and explanation. Genome Biol 2011; 12:R60.

35. Kanehisa M, Goto S, Sato Y, Kawashima M, Furumichi M, Tanabe M. Data, information, knowledge and principle: back to metabolism in KEGG. Nucleic Acids Res 2014; 42:D199-205.

36. Powell S, Forslund K, Szklarczyk D, Trachana K, Roth A, Huerta-Cepas J, et al. eggNOG v4.0: nested orthology inference across 3686 organisms. Nucleic Acids Res 2014; 42:D231-9.

37. Cantarel BL, Coutinho PM, Rancurel C, Bernard T, Lombard V, Henrissat B. The Carbohydrate-Active EnZymes database (CAZy): an expert resource for Glycogenomics. Nucleic Acids Res 2009; 37:D233-8.

38. Backhed F, Roswall J, Peng Y, Feng Q, Jia H, Kovatcheva-Datchary P, et al. Dynamics and Stabilization of the Human Gut Microbiome during the First Year of Life. Cell Host Microbe 2015; 17:690-703.

39. Martinez JL, Coque TM, Baquero F. What is a resistance gene? Ranking risk in resistomes. Nat Rev Microbiol 2015; 13:116-23.
