# Supplementary figures and images for: High-sugar, high-fat, and high-protein diets promote antibiotic resistance gene spreading in the mouse intestinal microbiota
Source: Gut Microbes. 2022 Jan 14;14(1):2022442. doi: 10.1080/19490976.2021.2022442 (PMC8765071; doi:10.1080/19490976.2021.2022442)

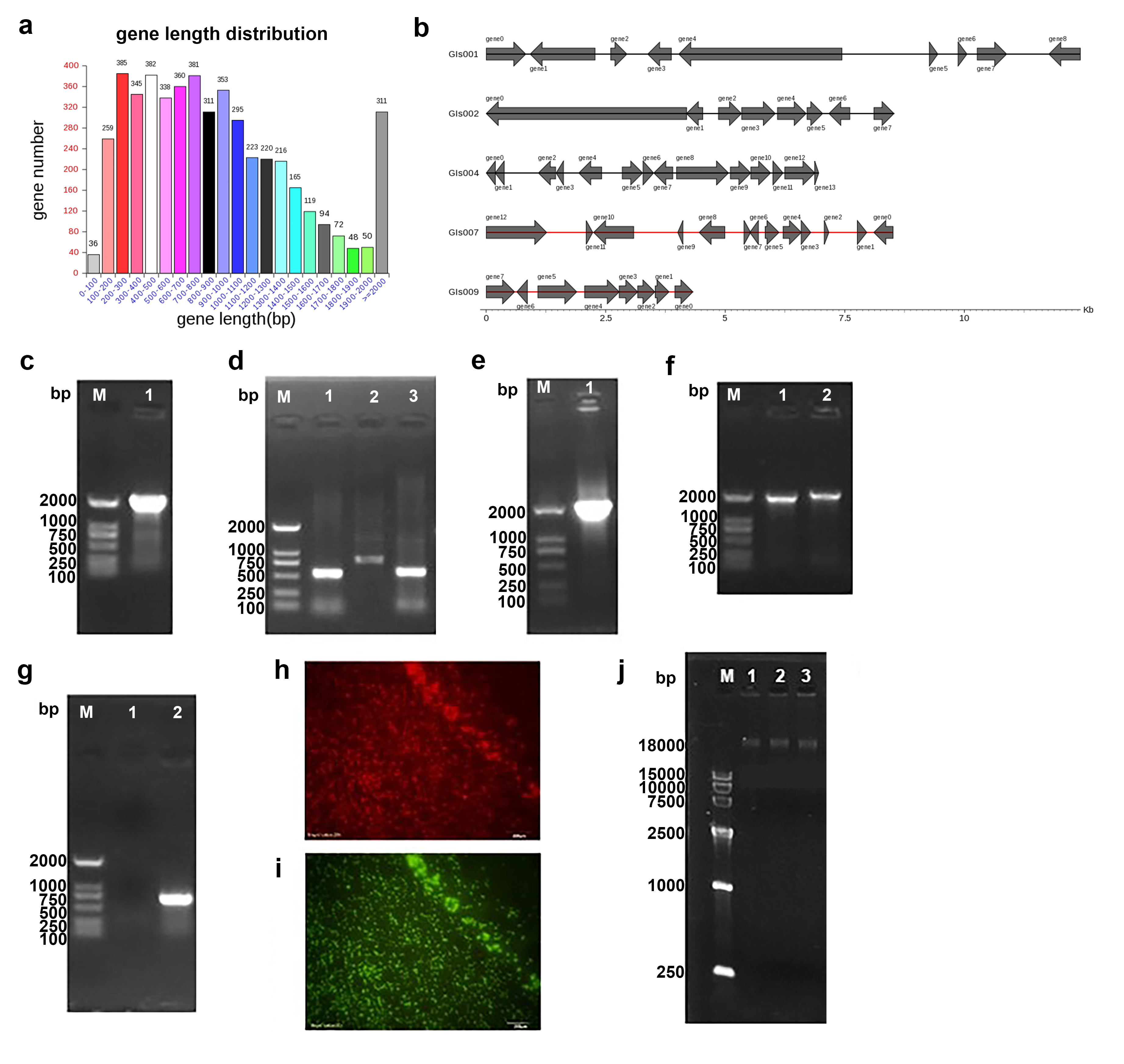

Supplement: Supplemental Material [file KGMI_A_2022442_SM9719.zip › supplementary/Supplementary Fig1.jpg]

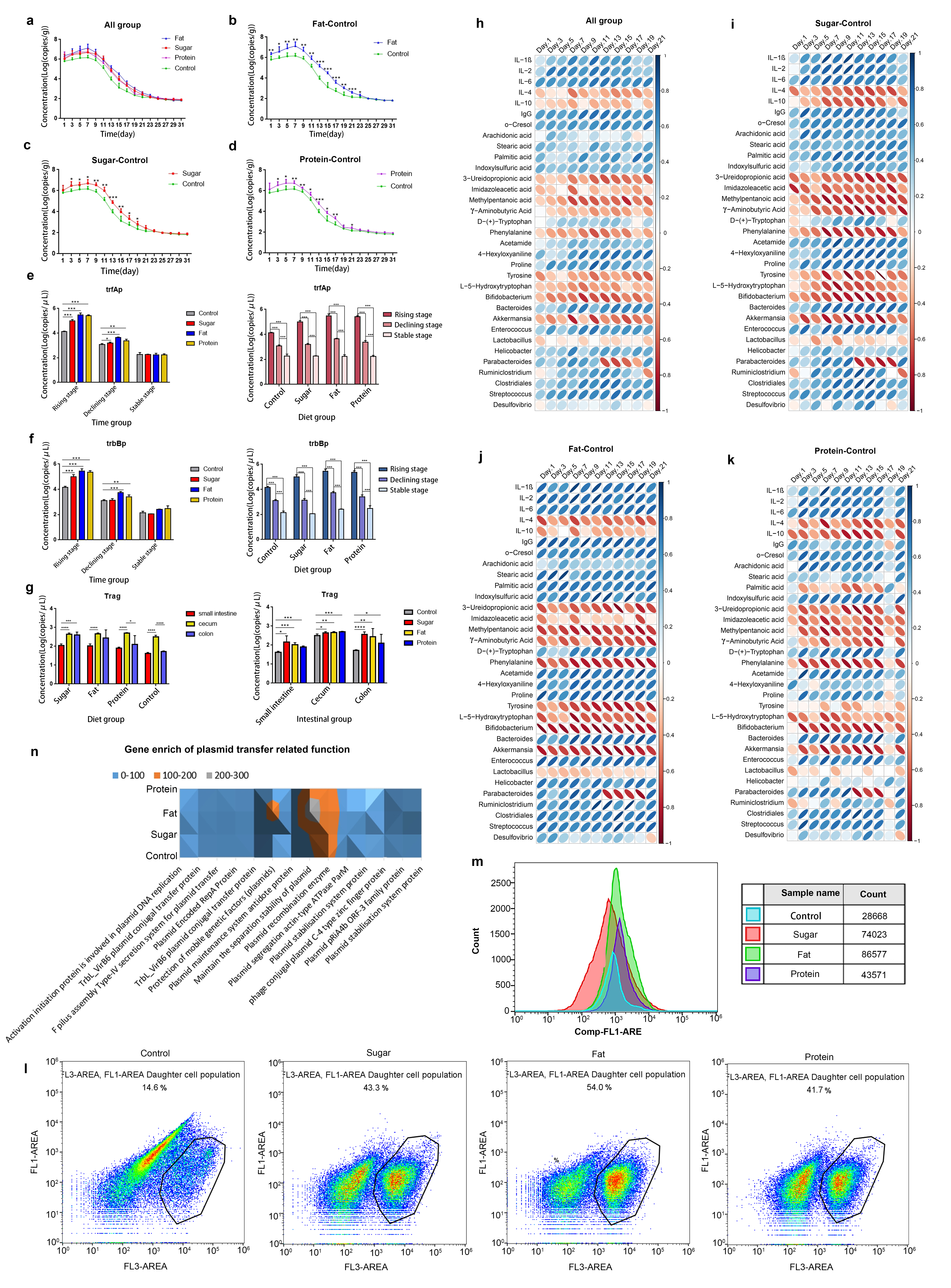

Supplement: Supplemental Material [file KGMI_A_2022442_SM9719.zip › supplementary/Supplementary Fig2.jpg]

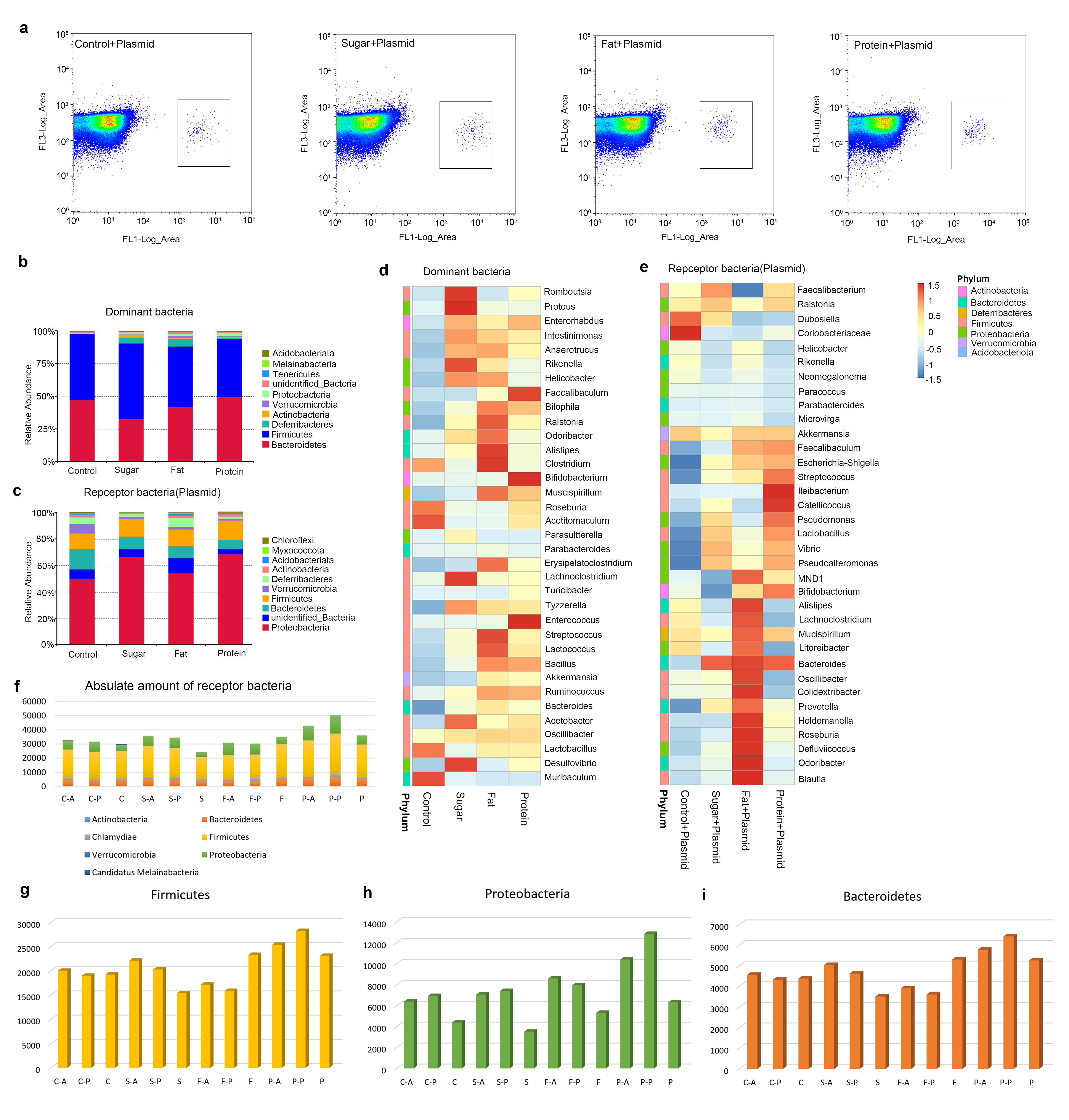

Supplement: Supplemental Material [file KGMI_A_2022442_SM9719.zip › supplementary/Supplementary Fig3.jpg]
